# Development and validation of algorithms to classify type 1 and 2 diabetes according to age at diagnosis using electronic health records

**DOI:** 10.1186/s12874-020-00921-3

**Published:** 2020-02-24

**Authors:** Calvin Ke, Thérèse A. Stukel, Andrea Luk, Baiju R. Shah, Prabhat Jha, Eric Lau, Ronald C. W. Ma, Wing-Yee So, Alice P. Kong, Elaine Chow, Juliana C. N. Chan

**Affiliations:** 1 Department of Medicine and Therapeutics, The Chinese University of Hong Kong, Prince of Wales Hospital, Shatin, Hong Kong; 2 Department of Medicine, University of Toronto, Toronto, Canada; 3 Institute of Health Policy, Management and Evaluation, University of Toronto, Toronto, Canada; 4 ICES, Toronto, Canada; 5 Asia Diabetes Foundation, Prince of Wales Hospital, Shatin, Hong Kong; 6 Hong Kong Institute of Diabetes and Obesity, The Chinese University of Hong Kong, Prince of Wales Hospital, Shatin, Hong Kong; 7 Li Ka Shing Institute of Health Science, The Chinese University of Hong Kong, Prince of Wales Hospital, Shatin, Hong Kong; 8 Department of Medicine, Sunnybrook Health Sciences Centre, Toronto, Canada; 9 Centre for Global Health Research, St. Michael’s Hospital, and Dalla Lana School of Public Health, University of Toronto, Toronto, Canada

**Keywords:** Validation study, Type 1 diabetes, Type 2 diabetes, Chinese ethnicity, Electronic health records, Administrative data, Population-based study, Health services research

## Abstract

**Background:**

Validated algorithms to classify type 1 and 2 diabetes (T1D, T2D) are mostly limited to white pediatric populations. We conducted a large study in Hong Kong among children and adults with diabetes to develop and validate algorithms using electronic health records (EHRs) to classify diabetes type against clinical assessment as the reference standard, and to evaluate performance by age at diagnosis.

**Methods:**

We included all people with diabetes (age at diagnosis 1.5–100 years during 2002–15) in the Hong Kong Diabetes Register and randomized them to derivation and validation cohorts. We developed candidate algorithms to identify diabetes types using encounter codes, prescriptions, and combinations of these criteria (“combination algorithms”). We identified 3 algorithms with the highest sensitivity, positive predictive value (PPV), and kappa coefficient, and evaluated performance by age at diagnosis in the validation cohort.

**Results:**

There were 10,196 (T1D *n* = 60, T2D *n* = 10,136) and 5101 (T1D *n* = 43, T2D *n* = 5058) people in the derivation and validation cohorts (mean age at diagnosis 22.7, 55.9 years; 53.3, 43.9% female; for T1D and T2D respectively). Algorithms using codes or prescriptions classified T1D well for age at diagnosis < 20 years, but sensitivity and PPV dropped for older ages at diagnosis. Combination algorithms maximized sensitivity or PPV, but not both. The “high sensitivity for type 1” algorithm (ratio of type 1 to type 2 codes ≥ 4, or at least 1 insulin prescription within 90 days) had a sensitivity of 95.3% (95% confidence interval 84.2–99.4%; PPV 12.8%, 9.3–16.9%), while the “high PPV for type 1” algorithm (ratio of type 1 to type 2 codes ≥ 4, and multiple daily injections with no other glucose-lowering medication prescription) had a PPV of 100.0% (79.4–100.0%; sensitivity 37.2%, 23.0–53.3%), and the “optimized” algorithm (ratio of type 1 to type 2 codes ≥ 4, and at least 1 insulin prescription within 90 days) had a sensitivity of 65.1% (49.1–79.0%) and PPV of 75.7% (58.8–88.2%) across all ages. Accuracy of T2D classification was high for all algorithms.

**Conclusions:**

Our validated set of algorithms accurately classifies T1D and T2D using EHRs for Hong Kong residents enrolled in a diabetes register. The choice of algorithm should be tailored to the unique requirements of each study question.

## Background

Administrative health databases are an important resource for population-based diabetes research [1]. Using routinely-collected data such as billing codes and hospitalization records, various algorithms have been developed to identify diabetes [2, 3]. While these algorithms capture diabetes diagnoses, they cannot accurately identify diabetes type [2–5]. Type 1 diabetes (T1D) is an autoimmune disease that classically occurs in children, but may rarely occur in older adults [6]. In T1D, autoantibodies destroy the insulin-producing pancreatic beta cells, causing insulin deficiency and hyperglycemia. Type 2 diabetes (T2D), which typically occurs in adulthood, is caused by genetic and other risk factors such as obesity that lead to insulin resistance and hyperglycemia, although lean individuals may also develop T2D due to insulin deficiency [6]. While T1D must be treated with insulin, T2D may be treated with lifestyle modification, insulin, or other glucose-lowering medications [6].

Many epidemiological studies apply the untested assumption that findings in adults with diabetes are representative of T2D [7, 8]. However, the prognoses of T1D and T2D are markedly different [9]—especially among adults aged < 40 years, where both types commonly occur and may be difficult to distinguish clinically [1, 9]. In this age group, it has been shown that T2D is associated with a 15-fold elevation in the risk of cardiovascular complications versus T1D [9]. Yet, diabetes types are poorly documented in administrative databases, which were not originally designed for research purposes. Specific diagnostic codes for T1D and T2D may be erroneously entered [10] or unavailable in some billing systems [2]. Furthermore, classification of diabetes type is particularly important in Asia because disaggregated population-level T1D and T2D incidence and prevalence have never been measured [11].

Considering the lifelong and immediate need for insulin treatment in T1D, novel algorithms have been developed to identify T1D using prescriptions and laboratory data from electronic health records (EHRs) [12]. However, previous validation studies had small sample sizes and were mostly limited to children in white populations [13–16]. One study developed and validated a complex algorithm to detect T1D in a US population with 65% (36–100%) sensitivity and 88% (78–98%) positive predictive value (PPV) using EHRs [12]. However, algorithms developed for white populations may have a poorer PPV when applied to Asian populations, as the prevalence of T1D in Asians appears to be much lower than white people [17]. The proportion of diabetes cases classified as T1D and T2D also varies enormously by age at diagnosis; yet, the effect of age at diagnosis on the performance of classification algorithms has never been specifically studied. To address these gaps, we conducted a large study among Hong Kong residents with diabetes to develop and validate algorithms using EHRs to classify T1D and T2D against clinical assessment as the reference standard, and to evaluate performance by age at diagnosis.

## Methods

### Setting and data sources

Hong Kong is a special administrative region of China with a population of 7.3 million and an estimated diabetes prevalence of 10.3% (2014) [18]. All residents are entitled to universal inpatient and outpatient health services operated by the governmental Hong Kong Hospital Authority (HA), which is modeled after the National Health Service of Britain. Given the wide public-private healthcare cost differential, HA hospitals account for about 95% of all bed-days [19].

The Hong Kong Diabetes Surveillance Database (HKDSD) includes all Hong Kong residents with diabetes as identified using the HA’s territory-wide EHR, which includes routinely-collected data on laboratory tests, prescriptions, and hospital visits for the entire population. We defined diabetes onset as the first occurrence of glycated haemoglobin A_1c_ ≥ 6.5% [20], fasting plasma glucose ≥ 7 mmol/L [21], glucose-lowering medication prescription [3, 4] excluding insulin, or long-term insulin prescription (≥ 28 days). To avoid detecting gestational diabetes [22], we excluded events occurring within 9 months prior to or 6 months after delivery (*International Statistical Classification of Diseases and Related Health Problems* version 9 (ICD-9) codes 72–75), or within 9 months of any pregnancy-related encounter (ICD-9 codes 630–676) outside these periods (in case of aborted pregnancies or delivery in a non-HA hospital). We also excluded in-patient glucose measurements to avoid misidentifying acute stress hyperglycemia as diabetes.

A subset of those in the HKDSD is additionally enrolled in the multicentre Hong Kong Diabetes Register (HKDR, Supplementary Table 1, Additional File). This register was established in 1995 at the Diabetes and Endocrine Centre at the Prince of Wales Hospital, a tertiary care public hospital in the New Territories East region with a catchment of 1.3 million residents, and was later expanded to 2 additional hospitals [23, 24]. Anyone with diabetes is eligible for enrolment in the HKDR. Referrals are self-initiated or from physicians located typically in community- or hospital-based clinics. All enrolled individuals undergo a comprehensive assessment including a detailed clinical history, fundoscopy and foot exams, and serum and urinary laboratory testing. This assessment yields detailed data including diabetes type, which is otherwise unavailable in the HKDSD. The research was approved by the Chinese University of Hong Kong–New Territories East Cluster Clinical Research Ethics Committee.

### Study population

**Fig. 1:**
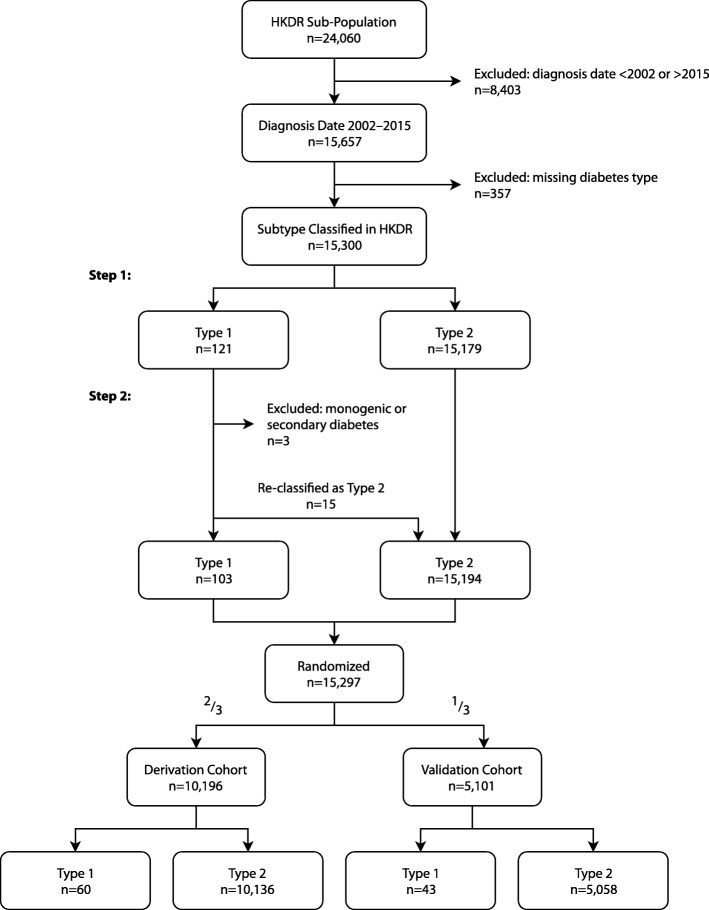
Flow diagram depicting creation of the study cohorts using the sub-population of people in the Hong Kong Diabetes Surveillance Database who were also enrolled in the Hong Kong Diabetes Register (HKDR). Diabetes type classification consisted of 2 steps: (1) comprehensive assessment, and (2) chart review of cases initially flagged as type 1 diabetes

Because the reference standard (clinical assessment) was only established for the subset of those enrolled in the HKDR, we restricted the study to this sub-population. To ensure at least 1 year of follow-up data, we included all people with diabetes diagnosed at ages 1.5 (to exclude neonatal diabetes) to 100 years from 1 January 2002 through 31 December 2015, defined using the HKDSD criteria. The maximum follow-up date was 31 December 2016. We excluded individuals with monogenic or secondary diabetes and those with missing diabetes type in the HKDR (Fig. 1). We randomized the remaining individuals into the derivation (two thirds) and validation (one third) cohorts.

### Reference standard

C-peptide and autoantibody testing are not routinely available to confirm T1D diagnosis in the public setting, and self-funded tests are rarely performed. Therefore, we applied the standard clinical definition of T1D adopted by the HKDR [25], which strictly defines T1D as diabetic ketoacidosis, unprovoked heavy ketones in urine or requirement of insulin within the first year of diagnosis. An endocrinologist reviewed all charts initially marked as T1D in the HKDR to ensure accuracy.

### Algorithm development and validation

We applied clinical knowledge (based on the experience of endocrinologists with expertise in diabetes management: CK, BRS, AL, JCNC) and reviewed previous validation studies [12–16, 26, 27] to develop candidate algorithms to identify T1D using either ICD-9 encounter codes (“code algorithms”; type 1 codes: 250.*x*1, 250.*x*3; type 2 codes: 250.*x*0, 250.*x*2) or prescriptions (“prescription algorithms”; Supplementary Tables 2–3, Additional File). We varied the number, ratio, and types of codes required, as well as the duration of time allowed between the diagnosis date and the initial insulin prescription. Positive cases were automatically classified as T1D and negative as T2D. Using the derivation cohort, we selected algorithms based on the sensitivity and PPV of identifying T1D, as these are the most important characteristics for public health [28]. Since the most sensitive algorithms had poor PPV and vice versa, we chose the best algorithms with the highest sensitivity and PPV separately, among both code and prescription algorithms (total: 4 algorithms, labelled A–D). We resolved ties by selecting the algorithm with the greatest sum of sensitivity and PPV. Then, we paired the 2 best code algorithms with the 2 best prescription algorithms using 2 methods in an effort to further improve accuracy [29, 30]. These methods were: combining using “or” (for example, “A *or* B”) to improve sensitivity, and combining using “and” (for example, “A *and* B”) to improve PPV. We then tested all 8 “combination algorithms” in the derivation cohort. Of the 12 code, prescription, and combination algorithms, we identified the 3 algorithms with the highest sensitivity, highest PPV, and highest kappa coefficient (“optimized” algorithm) across all ages. Using the validation cohort, we evaluated the performance of these 3 algorithms in classifying T1D and T2D by age at diagnosis.

We repeated the entire procedure using additional laboratory data (estimated glomerular filtration rate) to determine whether requiring normal renal function with insulin prescriptions would improve the performance of prescription algorithms.

### Statistical analysis

We calculated the sensitivity, specificity, PPV, and negative predictive value (NPV) with 95% exact confidence intervals of each selected algorithm for classifying T1D and T2D in the derivation and validation cohorts. We also calculated Cohen’s kappa coefficient, which represents agreement after agreement due to chance is removed [31]. A perfect algorithm would have sensitivity, specificity, PPV, and NPV values of 100%, and a kappa value of 1.0. Missing data were minimal (missing diabetes type: *n* = 357, 2.3%) and handled by complete case analysis. All analyses were performed using the “FREQ” procedure in SAS version 9.4 (Cary, NC).

## Results

There were 15,300 individuals with complete data and diabetes diagnosed during 2002–15 (Fig. 1). Of these cases, 121 were initially classified as T1D. After chart review, 3 were excluded as monogenic or secondary diabetes and 15 were re-classified as T2D, leaving 103 T1D cases remaining. The final cohorts consisted of 10,196 (derivation) and 5101 (validation) individuals. Tables 1 and 2 show the baseline demographic characteristics of the study cohorts. The distribution of baseline characteristics was highly similar across the derivation and validation cohorts and across the HKDR and HKDSD, although the HKDR population had more prescriptions for insulin and other glucose-lowering medications. The average age at diagnosis was 22.7 years for T1D and 55.9 years for T2D (Table 2; see Supplementary Figure 1, Additional File). More men (56.1%) had T2D, but for T1D the sex ratio was more balanced. People with T1D had a median of 3.0 type 1 codes, including 2.0 from the primary diagnosis on the hospital discharge abstract. People with T2D had a median of 1 type 2 code. Although most people with T1D had at least 1 type 1 code (83.3% sensitivity), the PPV for this algorithm was only 26.0%. Most people with T1D also had at least 1 type 2 code (70.0%). Code algorithms using a ratio of type 1 to type 2 codes had a higher PPV and similar sensitivity compared to those using the number of type 1 or type 2 codes. Two algorithms had the highest sensitivity (83.3%), but “ratio of type 1 to type 2 codes ≥ 0.5” (algorithm A) was chosen because it had a higher PPV (34.0%) than “at least 1 type 1 code.” “Ratio of type 1 to type 2 codes ≥ 4” (algorithm B) was chosen for having the highest PPV (57.3%, sensitivity 71.7%).

**Table 1:** Baseline characteristics of people in the Hong Kong Diabetes Register (HKDR, randomized 2:1 into derivation and validation cohorts) and the Hong Kong Diabetes Surveillance Database (HKDSD). Laboratory and prescription data are from the first year after diagnosis. Values are counts (*n*) and percentages unless otherwise indicated

	HKDR				HKDSD	
	Cohort		Total *n* = 15,297	Missing (*n*, %)	*n* = 561,924	Missing (*n*, %)
	Derivation *n* = 10,196	Validation *n* = 5101				
Age (years; mean, standard deviation)	55.7 (11.7)	55.6 (11.8)	55.7 (11.7)	0 (0.0)	61.8 (13.2)	0 (0.0)
Age < 18 years	36 (0.4)	28 (0.6)	64 (0.4)		1577 (0.3)	
Age 18–39 years	801 (7.9)	375 (7.4)	1176 (7.7)		24,148 (4.3)	
Age ≥ 40 years	9359 (91.8)	4698 (92.1)	14,057 (91.9)		536,199 (95.4)	
Female	4488 (44.0)	2341 (45.9)	6829 (44.6)		270,282 (48.1)	
Baseline Comorbidities*						
Ischemic heart disease	972 (9.5)	456 (8.9)	1428 (9.3)		49,931 (8.9)	
Congestive heart failure	411 (4.0)	196 (3.8)	607 (4.0)		28,745 (5.1)	
Stroke	855 (8.4)	426 (8.4)	1281 (8.4)		54,762 (9.8)	
Peripheral arterial disease	125 (1.2)	52 (1.0)	177 (1.2)		5241 (0.9)	
Cancer	1071 (10.5)	537 (10.5)	1608 (10.5)		63,510 (11.3)	
Chronic kidney disease^†^	1885 (18.5)	948 (18.6)	2833 (18.5)	1 (0.0)	125,274 (22.5)	6101 (0.0)
End-stage renal disease^†^	133 (1.3)	78 (1.5)	211 (1.4)		8274 (1.5)	
Risk Factors (mean, standard deviation unless otherwise indicated)						
A1C (%)	7.4 (1.0)	7.4 (1.0)	7.4 (1.0)	17 (0.1)	7.2 (1.1)	24,485 (4.4)
Fasting plasma glucose (mmol/L)	7.7 (1.7)	7.7 (1.7)	7.7 (1.7)	90 (0.6)	7.4 (1.8)	32,708 (5.8)
LDL-C (mmol/L)	2.5 (0.6)	2.5 (0.6)	2.5 (0.6)	48 (0.3)	2.7 (0.7)	38,691 (6.9)
HDL-C (mmol/L)	1.3 (0.3)	1.3 (0.3)	1.3 (0.3)	45 (0.3)	1.3 (0.3)	37,813 (6.7)
Triglycerides (median, IQR; mmol/L)	1.4 (0.9)	1.4 (0.9)	1.4 (0.9)	41 (0.3)	1.4 (0.9)	34,211 (6.1)
eGFR (mL/min/1.73 m^2^)	79.4 (23.3)	79.9 (23.5)	79.5 (23.4)	1 (0.0)	76.3 (23.4)	6101 (0.0)
Glucose-Lowering Medications (excluding insulin)						
Metformin	9044 (88.7)	4540 (89.0)	13,584 (88.8)		399,235 (71.0)	
Sulfonylureas	7492 (73.5)	3752 (73.6)	11,244 (73.5)		301,158 (53.6)	
Thiazolidinediones	674 (6.6)	306 (6.0)	980 (6.4)		11,414 (2.0)	
DPP-4 inhibitors	1945 (19.1)	932 (18.3)	2877 (18.8)		24,529 (4.4)	
GLP-1 agonists	32 (0.3)	21 (0.4)	53 (0.4)		319 (0.1)	
SGLT2 inhibitors	171 (1.7)	79 (1.6)	250 (1.6)		1381 (0.2)	
Alpha-glucosidase inhibitor	347 (3.4)	159 (3.1)	506 (3.3)		7885 (1.4)	
Insulin^‡^ (*n*, %)						
Long-acting	505 (5.0)	232 (4.6)	737 (4.8)		7260 (1.3)	
Intermediate-acting	2903 (28.5)	1397 (27.4)	4303 (28.1)		54,859 (9.8)	
Short-acting	1337 (13.1)	630 (12.4)	1967 (12.9)		58,096 (10.3)	
Premixed	907 (8.9)	430 (8.4)	1337 (8.7)		18,040 (3.2)	
Multiple daily injections**	1001 (9.8)	480 (9.4)	1481 (9.7)		23,445 (4.2)	
Any insulin	3454 (33.9)	1648 (32.3)	5102 (33.4)		94,974 (16.9)	
Other Medications						
Statin	7098 (69.6)	3546 (69.5)	10,644 (69.6)		349,324 (62.2)	
RAS inhibitor	7054 (69.2)	3530 (69.2)	10,584 (69.2)		344,949 (61.4)	
Antiplatelet agent	3651 (35.8)	1836 (36.0)	5487 (35.9)		197,612 (35.2)	

^*^based on principal diagnoses on hospitalization discharge abstracts within 2 years prior to diagnosis (except for renal conditions)

^†^chronic kidney disease eGFR< 60 mL/min/1.73 m^2^, end-stage renal disease eGFR< 15 mL/min/1.73 m^2^

^‡^prescriptions ≥ 28 days in duration

^**^any combination of long-acting and short-acting insulin

Abbreviations: *A1C* glycated haemoglobin A_1c_, *LDL-C* low-density lipoprotein cholesterol, *HDL-C* high-density lipoprotein cholesterol, *IQR* interquartile range, *eGFR* estimated glomerular filtration rate, *DPP-4* dipeptidyl peptidase-4, *GLP-1* glucagon-like peptide-1, *SGLT2* sodium-glucose transport protein 2, *RAS* renin-angiotensin system

**Table 2:** Baseline characteristics and performance of candidate algorithms among people in the derivation cohort, stratified by diabetes type. Candidate algorithms developed using encounter codes (“code algorithms”) or prescriptions (“prescription algorithms”) are also shown. For each algorithm, values in the Type 1 and 2 columns indicate the number and percentage of individuals satisfying the algorithm (sensitivity). Positive predictive values for classifying type 1 diabetes are shown in the right column. The best 4 algorithms are indicated by the letters in parentheses (A–D; see text for selection criteria)

	Type 1 (*n* = 60)	Type 2 (*n* = 10,136)	Positive Predictive Value (%)
Demographic Characteristics			
Age at diagnosis (years; mean, standard deviation)	22.7 (12.6)	55.9 (11.4)	
< 18	16 (26.7)	20 (0.2)	
18–39	33 (55.0)	768 (7.6)	
≥ 40 years	11 (18.3)	9348 (92.3)	
Female	32 (53.3)	4456 (43.9)	
Coding Characteristics*			
Number of type 1 codes (median, interquartile range)	3.0 (4.0)	0.0 (0.0)	
Total number of type 1principal codes (median, interquartile range)	2.0 (2.5)	0.0 (0.0)	
Total number of type 1 mixed codes (median, interquartile range)	1.0 (2.0)	0.0 (0.0)	
Number of type 2 codes (median, interquartile range)	0.0 (1.0)	1.0 (3.0)	
Total number of type 2 principal codes (median, interquartile range)	0.0 (0.0)	1.0 (2.0)	
Total number of type 2 mixed codes (median, interquartile range)	0.0 (0.0)	1.0 (2.0)	
Candidate Code Algorithms*			
At least 1 type 1 code	50 (83.3)	142 (1.4)	26.0
At least 1 type 1 principal code	42 (70.0)	65 (0.6)	39.2
At least 1 type 1 mixed code	38 (63.3)	108 (1.1)	26.0
At least 1 type 2 code	43 (71.7)	92 (0.9)	31.8
At least 1 type 2 principal code	29 (48.3)	25 (0.2)	53.7
At least 1 type 2 mixed code	21 (35.0)	66 (0.6)	24.1
Ratio of type 1 to type 2 codes ≥ 0.5 (A)	50 (83.3)	97 (1.0)	34.0
Ratio of type 1 to type 2 codes ≥ 0.75	49 (81.7)	81 (0.8)	37.7
Ratio of type 1 to type 2 codes ≥ 1	49 (81.7)	78 (0.8)	38.6
Ratio of type 1 to type 2 codes ≥ 2	47 (78.3)	47 (0.5)	50.0
Ratio of type 1 to type 2 codes ≥ 3	46 (76.7)	38 (0.4)	54.8
Ratio of type 1 to type 2 codes ≥ 4 (B)	43 (71.7)	32 (0.3)	57.3
Candidate Prescription Algorithms^†^			
At least 1 insulin prescription	59 (98.3)	3408 (33.6)	1.7
within 90 days (C)	58 (96.7)	615 (6.1)	8.6
within 180 days	58 (96.7)	715 (7.1)	7.5
within 365 days	58 (96.7)	844 (8.3)	6.4
At least 1 insulin prescription with no other glucose-lowering medication prescription	36 (60.0)	80 (0.8)	31.0
within 90 days	43 (71.7)	362 (3.6)	10.6
within 180 days	44 (73.3)	483 (4.8)	8.4
within 365 days	45 (75.0)	653 (6.4)	6.4
At least 1 insulin prescription with no other glucose-lowering medication prescription except metformin	50 (83.3)	275 (2.7)	15.4
within 90 days	53 (88.3)	451 (4.4)	10.5
within 180 days	54 (90.0)	566 (5.6)	8.7
within 365 days	54 (90.0)	727 (7.2)	6.9
Multiple daily injections^‡^	47 (78.3)	273 (2.7)	14.7
within 90 days	7 (11.7)	5 (0.0)	58.3
within 180 days	8 (13.3)	9 (0.1)	47.1
within 365 days	12 (20.0)	13 (0.1)	48.0
Multiple daily injections with no other glucose-lowering medication prescription (D)	32 (53.3)	9 (0.1)	78.0
within 90 days	7 (11.7)	2 (0.0)	77.8
within 180 days	8 (13.3)	6 (0.1)	571
within 365 days	12 (20.0)	11 (0.1)	52.2
Multiple daily injections with no other glucose-lowering medication prescription except metformin	42 (70.0)	26 (0.3)	61.8
within 90 days	7 (11.7)	2 (0.0)	77.8
within 180 days	8 (13.3)	6 (0.1)	57.1
within 365 days	12 (20.0)	11 (0.1)	52.2
At least 1 metformin prescription	22 (36.7)	8979 (88.6)	0.2
Other glucose-lowering medication prescription excluding insulin and metformin	10 (16.7)	7673 (75.7)	0.2

*In our dataset, encounter codes were classified as “principal” (principal diagnoses from hospital discharge abstracts) or “mixed” (including secondary diagnoses from hospital discharge abstracts and encounter codes from hospital outpatient clinics). Type 1 codes are defined as International Classifications of Diseases Ninth Revision (ICD-9) codes 250.*x*1 or 250.*x*3; type 2 codes are defined as ICD-9 codes 250.*x*0 or 250.*x*2

^†^We only included long-term insulin prescriptions (duration ≥ 28 days). See Appendix Table 4 for prescriptions algorithms using renal function criteria. All indicated durations are counted from the diagnosis date. If no duration is indicated, all data were utilized (up to 2016)

^‡^Multiple daily injections: defined as prescriptions for long-acting and short-acting insulin (both initiated within the same time frame as specified)

See Supplementary Table 4 (Additional File 1) for algorithms using renal function criteria

Among the prescription algorithms, those specifying “at least 1 insulin prescription” were the most sensitive but lacked PPV for classifying T1D. Nearly everyone with T1D received an insulin prescription at any time (59 of 60 people, 98.3% sensitivity), and almost all received it within 90 days of diabetes diagnosis (58 of 59 people, 96.7% sensitivity). As these 2 prescription algorithms had the highest sensitivity values and classified everyone identically except for 1 case, we applied the tiebreaker criteria to choose “insulin prescription within 90 days” (algorithm C) based on its greater PPV (8.6%, versus 1.7% for “insulin prescription at any time”). Adding criteria for other types of medications improved the PPV of insulin-based prescription algorithms at the expense of sensitivity. In the T1D cohort, 36.7% received at least 1 metformin prescription (versus 88.6% in the T2D cohort), and 16.7% received a glucose-lowering medication prescription other than insulin and metformin (versus 75.7% in the T2D cohort). Of the algorithms that added a condition for no other glucose-lowering medication prescriptions in addition to an insulin prescription, the algorithm “at least 1 insulin prescription with no other glucose-lowering medication prescriptions except for metformin” had the highest PPV (31.0%; sensitivity 60.0%). Specifying the type of insulin as multiple daily injections further improved the PPV. “Multiple daily injections with no other glucose-lowering medication prescription” (algorithm D) had a 78.0% PPV (sensitivity 53.3%), which was the highest of the prescription algorithms.

**Fig. 2:**
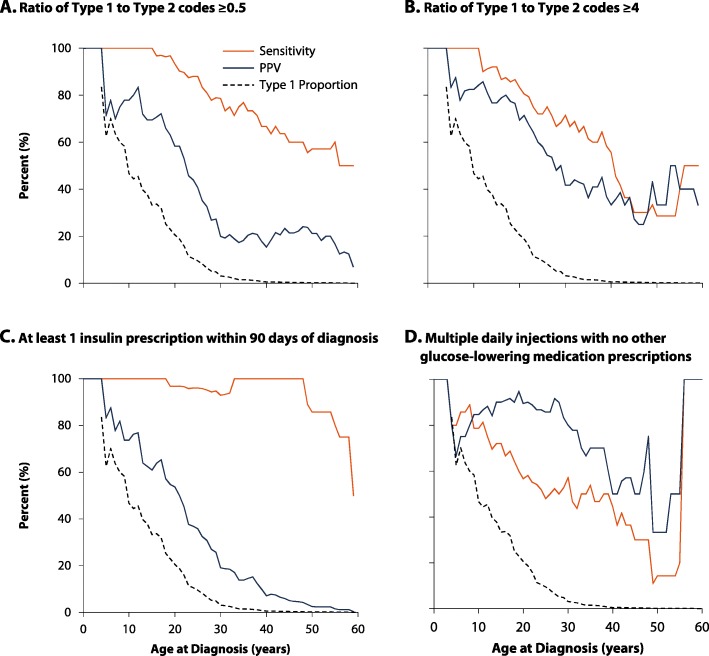
Sensitivity and positive predictive value of the 4 best single algorithms for classifying type 1 diabetes in the derivation cohort by age at diagnosis*, displayed with the proportion of all diabetes cases classified as type 1 using the reference standard (dashed line). Algorithms: (**a**) ratio of type 1 to type 2 codes ≥ 0.5; (**b**) ratio of type 1 to type 2 codes ≥ 4; (**c**) at least 1 insulin prescription within 90 days of diagnosis; (**d**) multiple daily injections with no other glucose-lowering medication prescriptions *smoothed using 15-year moving averages

Algorithms A–D classified T1D well for age at diagnosis < 20 years in the derivation cohort, but as the proportion of diabetes cases classified as T1D dropped with age, the precision and estimates of sensitivity and PPV also dropped (Fig. 2). For age at diagnosis < 20 years, algorithm B had the highest kappa coefficient (sensitivity: 91.3, 95% confidence interval 72.0–98.9%; PPV: 80.8%, 60.6–93.4%; Table 3). For age at diagnosis ≥ 20 years, algorithm C was the most sensitive but lacked PPV, while algorithm D had the highest PPV and kappa coefficient, despite a low sensitivity (age at diagnosis 20–39 years: sensitivity 50.0%, 29.9–70.1%, PPV 81.3, 54.4–96.0%; ≥ 40 years: sensitivity 27.3%, 6.0–61.0%, PPV 50.0%, 11.8–88.2%).

**Table 3:** Test characteristics of single (A–D) and combination algorithms for classifying type 1 diabetes compared to the reference standard in the derivation cohort, stratified by age at diagnosis. Sensitivity, specificity, positive predictive value (PPV) and negative predictive value (NPV) are percentages with 95% confidence intervals. Cohen’s kappa coefficient represents agreement after agreement due to chance is removed (1.0 indicates perfect agreement) [31]. The “Type 1 Proportion” columns refer to the percentage of people in the cohort with diabetes classified as having type 1 using each algorithm (“Calculated”) and the reference standard (“True”). The best overall algorithms are marked (* = highest sensitivity, ^†^ = highest PPV, ^‡^ = highest kappa coefficient)

Algorithm	TP	FP	FN	TN	Sensitivity	Specificity	PPV	NPV	Kappa	Type 1 Proportion (%)	
										Calculated	True
All Ages											
Ratio of type 1 to type 2 codes ≥ 0.5 (A)	50	97	10	10,039	83.3 (71.5, 91.7)	99.0 (98.8, 99.2)	34.0 (26.4, 42.3)	99.9 (99.8, 100.0)	0.48	1.4	0.6
Ratio of type 1 to type 2 codes ≥ 4 (B)	43	32	17	10,104	71.7 (58.6, 82.5)	99.7 (99.6, 99.8)	57.3 (45.4, 68.7)	99.8 (99.7, 99.9)	0.63	0.7	
At least 1 insulin prescription within 90 days (C)	58	615	2	9521	96.7 (88.5, 99.6)	93.9 (93.5, 94.4)	8.6 (6.6, 11.0)	100.0 (99.9, 100.0)	0.15	6.6	
Multiple daily injections with no other glucose-lowering medication prescription (D)	32	9	28	10,127	53.3 (40.0, 66.3)	99.9 (99.8, 100.0)	78.0 (62.4, 89.4)	99.7 (99.6, 99.8)	0.63	0.4	
A and C	49	52	11	10,084	81.7 (69.6, 90.5)	99.5 (99.3, 99.6)	48.5 (38.4, 58.7)	99.9 (99.8, 99.9)	0.61	1.0	
A and D	28	5	32	10,131	46.7 (33.7, 60.0)	100.0 (99.9, 100.0)	84.8 (68.1, 94.9)	99.7 (99.6, 99.8)	0.60	0.3	
B and C^‡^	42	19	18	10,117	70.0 (56.8, 81.2)	99.8 (99.7, 99.9)	68.9 (55.7, 80.1)	99.8 (99.7, 99.9)	0.69	0.6	
B and D^†^	25	4	35	10,132	41.7 (29.1, 55.1)	100.0 (99.9, 100.0)	86.2 (68.3, 96.1)	99.7 (99.5, 99.8)	0.56	0.3	
A or C	59	660	1	9476	98.3 (91.1, 100.0)	93.5 (93.0, 94.0)	8.2 (6.3, 10.5)	100.0 (99.9, 100.0)	0.14	7.1	
A or D	54	101	6	10,035	90.0 (79.5, 96.2)	99.0 (98.8, 99.2)	34.8 (27.4, 42.9)	99.9 (99.9, 100.0)	0.50	1.5	
B or C*	59	628	1	9508	98.3 (91.1, 100.0)	93.8 (93.3, 94.3)	8.6 (6.6, 10.9)	100.0 (99.9, 100.0)	0.15	6.7	
B or D	50	37	10	10,099	83.3 (71.5, 91.7)	99.6 (99.5, 99.7)	57.5 (46.4, 68.0)	99.9 (99.8, 100.0)	0.68	0.9	
Age < 20 years											
Ratio of type 1 to type 2 codes ≥ 0.5 (A)	23	9	0	26	100.0 (85.2, 100.0)	74.3 (56.7, 87.5)	71.9 (53.3, 86.3)	100.0 (86.8, 100.0)	0.70	55.2	39.7
Ratio of type 1 to type 2 codes ≥ 4 (B)	21	5	2	30	91.3 (72.0, 98.9)	85.7 (69.7, 95.2)	80.8 (60.6, 93.4)	93.8 (79.2, 99.2)	0.75	44.8	
At least 1 insulin prescription within 90 days (C)	23	13	0	22	100.0 (85.2, 100.0)	62.9 (44.9, 78.5)	63.9 (46.2, 79.2)	100.0 (84.6, 100.0)	0.57	62.1	
Multiple daily injections with no other glucose-lowering medication prescription (D)	16	3	7	32	69.6 (47.1, 86.8)	91.4 (76.9, 98.2)	84.2 (60.4, 96.6)	82.1 (66.5, 92.5)	0.63	32.8	
A and C	23	8	0	27	100.0 (85.2, 100.0)	77.1 (59.9, 89.6)	74.2 (55.4, 88.1)	100.0 (87.2, 100.0)	0.73	53.4	
A and D	16	2	7	33	69.6 (47.1, 86.8)	94.3 (80.8, 99.3)	88.9 (65.3, 98.6)	82.5 (67.2, 92.7)	0.66	31.0	
B and C	21	5	2	30	91.3 (72.0, 98.9)	85.7 (69.7, 95.2)	80.8 (60.6, 93.4)	93.8 (79.2, 99.2)	0.75	44.8	
B and D	15	2	8	33	65.2 (42.7, 83.6)	94.3 (80.8, 99.3)	88.2 (63.6, 98.5)	80.5 (65.1, 91.2)	0.62	29.3	
A or C	23	14	0	21	100.0 (85.2, 100.0)	60.0 (42.1, 76.1)	62.2 (44.8, 77.5)	100.0 (83.9, 100.0)	0.54	63.8	
A or D	23	10	0	25	100.0 (85.2, 100.0)	71.4 (53.7, 85.4)	69.7 (51.3, 84.4)	100.0 (86.3, 100.0)	0.66	56.9	
B or C	23	13	0	22	100.0 (85.2, 100.0)	62.9 (44.9, 78.5)	63.9 (46.2, 79.2)	100.0 (84.6, 100.0)	0.57	62.1	
B or D	22	6	1	29	95.7 (78.1, 99.9)	82.9 (66.4, 93.4)	78.6 (59.0, 91.7)	96.7 (82.8, 99.9)	0.76	48.3	
Age 20–39 years											
Ratio of type 1 to type 2 codes ≥ 0.5 (A)	21	53	5	700	80.8 (60.6, 93.4)	93.0 (90.9, 94.7)	28.4 (18.5, 40.1)	99.3 (98.4, 99.8)	0.39	9.5	3.3
Ratio of type 1 to type 2 codes ≥ 4 (B)	19	16	7	737	73.1 (52.2, 88.4)	97.9 (96.6, 98.8)	54.3 (36.6, 71.2)	99.1 (98.1, 99.6)	0.61	4.5	
At least 1 insulin prescription within 90 days (C)	25	80	1	673	96.2 (80.4, 99.9)	89.4 (87.0, 91.5)	23.8 (16.0, 33.1)	99.9 (99.2, 100.0)	0.35	13.5	
Multiple daily injections with no other glucose-lowering medication prescription (D)	13	3	13	750	50.0 (29.9, 70.1)	99.6 (98.8, 99.9)	81.3 (54.4, 96.0)	98.3 (97.1, 99.1)	0.61	2.1	
A and C	20	23	6	730	76.9 (56.4, 91.0)	96.9 (95.5, 98.1)	46.5 (31.2, 62.3)	99.2 (98.2, 99.7)	0.56	5.5	
A and D	10	1	16	752	38.5 (20.2, 59.4)	99.9 (99.3, 100.0)	90.9 (58.7, 99.8)	97.9 (96.6, 98.8)	0.53	1.4	
B and C	18	8	8	745	69.2 (48.2, 85.7)	98.9 (97.9, 99.5)	69.2 (48.2, 85.7)	98.9 (97.9, 99.5)	0.68	3.3	
B and D	10	1	16	752	38.5 (20.2, 59.4)	99.9 (99.3, 100.0)	90.9 (58.7, 99.8)	97.9 (96.6, 98.8)	0.53	1.4	
A or C	26	110	0	643	100.0 (86.8, 100.0)	85.4 (82.7, 87.8)	19.1 (12.9, 26.7)	100.0 (99.4, 100.0)	0.28	17.5	
A or D	24	55	2	698	92.3 (74.9, 99.1)	92.7 (90.6, 94.5)	30.4 (20.5, 41.8)	99.7 (99.0, 100.0)	0.43	10.1	
B or C	26	88	0	665	100.0 (86.8, 100.0)	88.3 (85.8, 90.5)	22.8 (15.5, 31.6)	100.0 (99.4, 100.0)	0.34	14.6	
B or D	22	18	4	735	84.6 (65.1, 95.6)	97.6 (96.2, 98.6)	55.0 (38.5, 70.7)	99.5 (98.6, 99.9)	0.65	5.1	
Age ≥ 40 years											
Ratio of type 1 to type 2 codes ≥ 0.5 (A)	6	35	5	9313	54.5 (23.4, 83.3)	99.6 (99.5, 99.7)	14.6 (5.6, 29.2)	99.9 (99.9, 100.0)	0.23	0.4	0.1
Ratio of type 1 to type 2 codes ≥ 4 (B)	3	11	8	9337	27.3 (6.0, 61.0)	99.9 (99.8, 99.9)	21.4 (4.7, 50.8)	99.9 (99.8, 100.0)	0.24	0.1	
At least 1 insulin prescription within 90 days (C)	10	522	1	8826	90.9 (58.7, 99.8)	94.4 (93.9, 94.9)	1.9 (0.9, 3.4)	100.0 (99.9, 100.0)	0.03	5.7	
Multiple daily injections with no other glucose-lowering medication prescription (D)	3	3	8	9345	27.3 (6.0, 61.0)	100.0 (99.9, 100.0)	50.0 (11.8, 88.2)	99.9 (99.8, 100.0)	0.35	0.1	
A and C	6	21	5	9327	54.5 (23.4, 83.3)	99.8 (99.7, 99.9)	22.2 (8.6, 42.3)	99.9 (99.9, 100.0)	0.31	0.3	
A and D	2	2	9	9346	18.2 (2.3, 51.8)	100.0 (99.9, 100.0)	50.0 (6.8, 93.2)	99.9 (99.8, 100.0)	0.27	0.0	
B and C	3	6	8	9342	27.3 (6.0, 61.0)	99.9 (99.9, 100.0)	33.3 (7.5, 70.1)	99.9 (99.8, 100.0)	0.30	0.1	
B and D	0	1	11	9347	0.0 (0.0, 28.5)	100.0 (99.9, 100.0)	Undefined	99.9 (99.8, 99.9)	0.00	0.0	
A or C	10	536	1	8812	90.9 (58.7, 99.8)	94.3 (93.8, 94.7)	1.8 (0.9, 3.3)	100.0 (99.9, 100.0)	0.03	5.8	
A or D	7	36	4	9312	63.6 (30.8, 89.1)	99.6 (99.5, 99.7)	16.3 (6.8, 30.7)	100.0 (99.9, 100.0)	0.26	0.5	
B or C	10	527	1	8821	90.9 (58.7, 99.8)	94.4 (93.9, 94.8)	1.9 (0.9, 3.4)	100.0 (99.9, 100.0)	0.03	5.7	
B or D	6	13	5	9335	54.5 (23.4, 83.3)	99.9 (99.8, 99.9)	31.6 (12.6, 56.6)	99.9 (99.9, 100.0)	0.40	0.2	

Abbreviations: *TP* true positive, *FP* false positive, *FN* false negative, *TN* true negative

If there were no true positive cases identified, the positive predictive was indicated as “undefined”

See Supplementary Table 5 (Additional File 1) for algorithms using renal function criteria

**Fig. 3:**
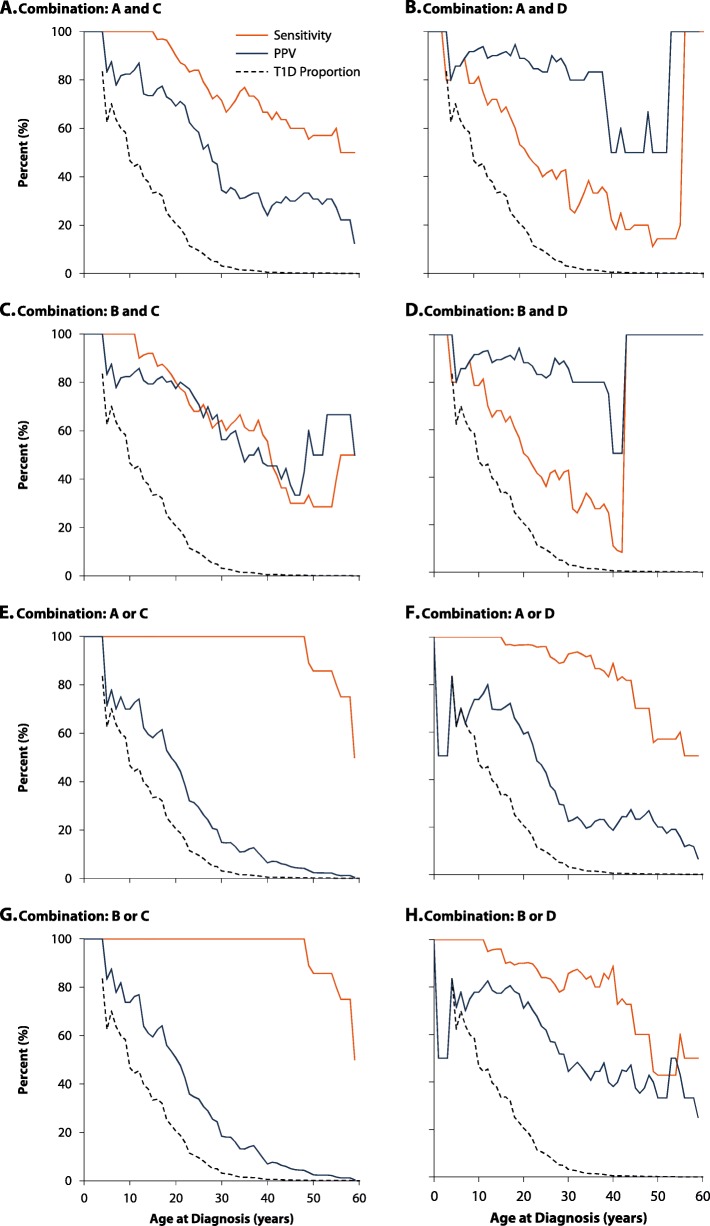
Sensitivity and positive predictive value of the 8 combination algorithms for classifying type 1 diabetes in the derivation cohort by age at diagnosis.* We paired single algorithms using “and” to maximize positive predictive value (panels **a**–**d**) and “or” to maximize sensitivity (panels **e**–**h**). See Fig. 2 for algorithm descriptions *smoothed using 15-year moving averages

As with algorithms A–D, performance of the combination algorithms also generally dropped at older ages at diagnosis (Fig. 3). For ages at diagnosis < 20 years, 4 combinations had 100.0% (85.2–100.0%; Table 3) sensitivity; among these algorithms, combination “A and C” had the highest PPV (74.2%, 55.4–88.1%). Among adults aged ≥ 20 years, sensitivity and PPV differed depending on the type of combination. “And” combinations had the highest PPV. “A and D” had the highest PPV among adults (age at diagnosis 20–39 years: 90.9%, 58.7–99.8%; ≥ 40 years: 50.0%, 11.8–88.2%), but the sensitivity was low (age at diagnosis 20–39 years: 38.5%, 20.2–59.4%, ≥40 years: 27.3%, 6.0–61.0%). Combinations “A or C” and “B or C” had the highest sensitivity (100.0%, 86.8–100.0%), while “B or C” had a relatively higher PPV (age at diagnosis 20–39 years: 38.5, 22.8%, 15.5–31.6%, ≥ 40 years: 1.9%, 0.9–3.4%). Among the “or” combinations, “A or C” and “B or C” had the identically highest sensitivity for classifying T1D (age at diagnosis 20–39 years: 100.0%, 86.8–100.0%, ≥ 40 years: 90.9%, 58.7–99.8%). However, these algorithms had low PPV (age at diagnosis 20–39 years: 19.1–22.8%, ≥ 40 years: 1.8–1.9%).

**Fig. 4:**
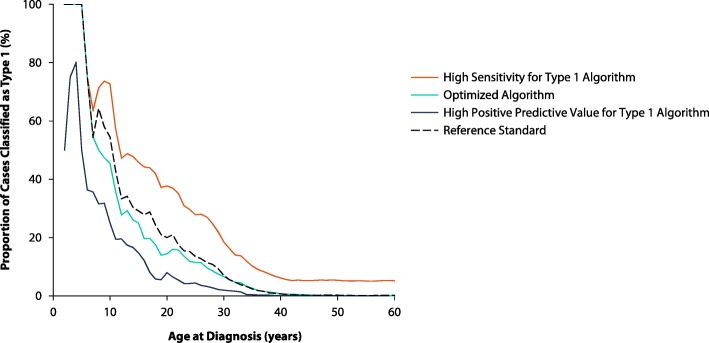
Proportion of all diabetes cases classified as type 1 by age at diagnosis in the validation cohort.* This proportion is calculated as the percentage of people in the cohort with diabetes classified as type 1 using the reference standard (dashed line), as well as high sensitivity for type 1, optimized, and high positive predictive value for type 1 algorithms (see Table 4 for descriptions) *smoothed using 15-year moving averages

Among the 12 algorithms we tested, “B or C,” “B and D,” and “B and C” had the best sensitivity (“high sensitivity for type 1” algorithm), PPV (“high PPV for type 1” algorithm), and kappa coefficient (“optimized” algorithm) respectively across all ages in the derivation cohort. Table 4 displays the performance characteristics of these algorithms in the validation cohort. The “high sensitivity for type 1” algorithm had a sensitivity of 95.3% (84.2–99.4%; PPV 12.8%, 9.3–16.9%), while the “high PPV for type 1” algorithm had a PPV of 100.0% (79.4–100.0%; sensitivity 37.2%, 23.0–53.3%) across all ages. The optimized algorithm had a sensitivity of 65.1% (49.1–79.0%) and PPV of 75.7% (58.8–88.2%) across all ages. These algorithms produced distinctive estimates of the proportion of cases classified as T1D among all diabetes cases according to age at diagnosis (Fig. 4). The high “PPV for type 1” algorithm yielded conservative estimates, while the “high sensitivity for type 1” algorithm inflated estimates. Estimates from “optimized” algorithm closely matched the reference standard across age at diagnosis.

**Table 4:** Test characteristics of the high sensitivity, high positive predictive value (PPV), and balanced algorithms for classifying type 1 diabetes compared to the reference standard in the validation cohort, stratified by age at diagnosis. Sensitivity, specificity, PPV and negative predictive value (NPV) are percentages with 95% confidence intervals. Cohen’s kappa coefficient represents agreement after agreement due to chance is removed (1.0 indicates perfect agreement) [31]. The “Type 1 Proportion” columns refer to the percentage of people in the cohort with diabetes classified as type 1 using each algorithm (“Calculated”) and the reference standard (“True”)

Algorithm	TP	FP	FN	TN	Sensitivity	Specificity	PPV	NPV	Kappa	Type 1 Proportion (%)	
										Calculated	True
High Sensitivity for Type 1: ratio of type 1 to type 2 codes ≥ 4, or at least 1 insulin prescription within 90 days											
All Ages	41	280	2	4778	95.3 (84.2, 99.4)	94.5 (93.8, 95.1)	12.8 (9.3, 16.9)	100.0 (99.8, 100.0)	0.21	6.3	0.8
Age < 20 years	14	6	0	21	100.0 (76.8, 100.0)	77.8 (57.7, 91.4)	70.0 (45.7, 88.1)	100.0 (83.9, 100.0)	0.71	48.8	34.1
Age 20–39 years	19	41	2	300	90.5 (69.6, 98.8)	88.0 (84.0, 91.2)	31.7 (20.3, 45.0)	99.3 (97.6, 99.9)	0.42	16.6	5.8
Age ≥ 40 years	8	233	0	4457	100.0 (63.1, 100.0)	95.0 (94.4, 95.6)	3.3 (1.4, 6.4)	100.0 (99.9, 100.0)	0.06	5.1	0.2
High PPV for Type 1: ratio of type 1 to type 2 codes ≥ 4, and multiple daily injections* with no other glucose-lowering medication prescription											
All Ages	16	0	27	5058	37.2 (23.0, 53.3)	100.0 (99.9, 100.0)	100.0 (79.4, 100.0)	99.5 (99.2, 99.6)	0.54	0.3	0.8
Age < 20 years	8	0	6	27	57.1 (28.9, 82.3)	100.0 (87.2, 100.0)	100.0 (63.1, 100.0)	81.8 (64.5, 93.0)	0.64	19.5	34.1
Age 20–39 years	6	0	15	341	28.6 (11.3, 52.2)	100.0 (98.9, 100.0)	100.0 (54.1, 100.0)	95.8 (93.1, 97.6)	0.43	1.7	5.8
Age ≥ 40 years	2	0	6	4690	25.0 (3.2, 65.1)	100.0 (99.9, 100.0)	100.0 (15.8, 100.0)	99.9 (99.7, 100.0)	0.40	0.0	0.2
Optimized: ratio of type 1 to type 2 codes ≥ 4, and at least 1 insulin prescription within 90 days											
All Ages	28	9	15	5049	65.1 (49.1, 79.0)	99.8 (99.7, 99.9)	75.7 (58.8, 88.2)	99.7 (99.5, 99.8)	0.70	0.7	0.8
Age < 20 years	12	0	2	27	85.7 (57.2, 98.2)	100.0 (87.2, 100.0)	100.0 (73.5, 100.0)	93.1 (77.2, 99.2)	0.89	29.3	34.1
Age 20–39 years	12	7	9	334	57.1 (34.0, 78.2)	97.9 (95.8, 99.2)	63.2 (38.4, 83.7)	97.4 (95.1, 98.8)	0.58	5.2	5.8
Age ≥ 40 years	4	2	4	4688	50.0 (15.7, 84.3)	100.0 (99.8, 100.0)	66.7 (22.3, 95.7)	99.9 (99.8, 100.0)	0.57	0.1	0.2

Abbreviations: *TP* true positive, *FP* false positive, *FN* false negative, *TN* true negative

*Multiple daily injections: defined as prescriptions for long-acting and short-acting insulin initiated in the same month

See Appendix Table 6 for algorithms using renal function criteria

Modifying algorithms with renal function criteria resulted in similar PPV with the same or lower sensitivity, and ultimately did not improve performance (Supplementary Tables 4–6, Additional File). All selected algorithms had high sensitivity and PPV in classifying T2D across all ages at diagnosis (sensitivity range 93.5–100.0%, PPV range 99.7–100.0%, Supplementary Table 7, Additional File). As all cases were classified as T1D or T2D in a binary fashion, the “high sensitivity for type 1” algorithm was equivalent to a “high PPV for type 2” algorithm, while the “high PPV for type 1” algorithm was equivalent to a “high sensitivity for type 2” algorithm (Supplementary Table 8, Additional File).

## Discussion

This is one of the largest validation studies of algorithms using EHRs to classify T1D and T2D among children and adults, and the only validation study in an Asian population. Using a systematic approach to generate a set of algorithms maximizing sensitivity and PPV, we revealed that classification performance is best at lower ages at diagnosis and drops as age at diagnosis increases—a finding that has not previously been demonstrated. We developed a “high sensitivity for type 1” algorithm (ratio of type 1 to type 2 codes ≥ 4, or at least 1 insulin prescription within 90 days) with > 90% sensitivity across age at diagnosis at the expense of lower PPV, and a “high PPV for type 1” algorithm (ratio of type 1 to type 2 codes ≥ 4, and multiple daily injections with no other glucose-lowering medication prescription) with perfect PPV across age at diagnosis at the expense of lower sensitivity. Our optimized algorithm (ratio of type 1 to type 2 codes ≥ 4, and at least 1 insulin prescription within 90 days) produced the most accurate estimates of the proportion of T1D cases across all ages at diagnosis. The complementary performance characteristics of these algorithms can inform their application to future studies, and the choice of algorithm should be tailored to the unique requirements of each study question.

Among children and adolescents, our diabetes classification algorithms performed similarly to others developed in white populations. Using Canadian administrative and prescription data, Vanderloo et al. [14] validated 4 algorithms using a combination of “Status Indian” registration, age < 10 years, and prescriptions to classify diabetes types. Although the sensitivity and PPV for classifying T1D were high (range: 96.9–99.2%), performance for identifying T2D was worse (sensitivity range: 55.4–84.2%; PPV range: 54.7–73.7%) and relied on ethnicity criteria that are not applicable in other populations. In a post-hoc analysis, we modified these algorithms by excluding inapplicable criteria and applied them to our data (Supplementary Tables 9–10, Additional File). These modified algorithms performed identically to our “high sensitivity for type 1” algorithm in classifying T1D (sensitivity 100.0%, 76.8–100.0%; PPV 70.0%, 45.7–88.1%) and T2D (sensitivity 77.8%, 57.7–91.4%; PPV 100.0%, 83.9–100.0%). In the large United States SEARCH for Diabetes in Youth Study (SEARCH), several algorithms were developed to identify diabetes type [13, 15, 16]. The “at least 1 outpatient T1D code” (sensitivity 94.8%, PPV 98.0% in SEARCH) [13] had 100.0% sensitivity (76.8–100.0%) and a better PPV (87.5%, 61.7–98.4%) than our “high sensitivity for type 1” algorithm. Other published SEARCH algorithms requiring the ratio of type 1 to total codes > 0.5 [15] and 0.6 [16] performed identically to our optimized algorithm (sensitivity 85.7–100.0%, PPV 87.5–100.0% for identifying T1D), although the latter algorithm required manual review to assess diabetes type for over a third of cases. The reasonable performance of these other algorithms confirms that T1D can be identified among children and adolescents using administrative and EHR data across different settings. Our results extend the literature with an expanded set of algorithms with optimal, maximally sensitive, or maximally predictive characteristics without the use of manual review, which would be unfeasible for large population-based studies.

By contrast, classification accuracy of the algorithms was lower among adults versus children. Previous validation studies including adults are limited. Klompas et al. [12] used a large EHR including primary and specialty care providers to develop and validate a complex algorithm (type 1 to type 2 codes > 0.5 and prescription for glucagon, type 1 to type 2 codes > 0.5 with no oral hypoglycemic other than metformin, C-peptide negative, autoantibodies positive, or prescription for urine acetone test strips) that reported a 65% (36–100%) sensitivity and 88% (78–98%) PPV for T1D and 100% (99–100%) sensitivity and 95% (88–100%) PPV for T2D. A modified version of this algorithm excluding urine acetone test strips was later tested separately [27]. However, these studies are limited by the lack of “and” combinations, and the use of a weighted sampling strategy that could have inflated estimates of PPV [12, 27]. Although algorithm performance in adults was not specifically reported, our post-hoc analysis showed that the algorithm proposed by Klompas et al. [12] (adapted to fit our data; see Supplementary Tables 9–10, Additional File) had decreased sensitivity (62.5%, 24.5–91.5%) and PPV (26.3%, 9.1–51.2%) among adults aged ≥ 40 years at diagnosis versus people aged < 20 years at diagnosis (sensitivity 100.0%, 76.8–100.0%, PPV 93.3%, 68.1–99.8%). The performance of another algorithm developed within a general practice EHR in the UK [26] showed a similar pattern using our data, although the overall performance was worse than our algorithms (sensitivity 39.5%, 25.0–55.6%; PPV 40.5%, 25.6–56.7% at all ages). While these results may be expected based on the rarity of T1D in adulthood, our large study adds a new approach to maximize sensitivity, PPV, or overall accuracy across all ages using different types of combinations. Moreover, we confirmed that renal function does not improve algorithm performance in adults, and this may reflect the growing variety of non-insulin agents available for people with diabetes and impaired renal function.

Our study yielded 3 complementary algorithms, the choice of which can be tailored to different study contexts depending on diabetes type, sensitivity, and PPV requirements. The optimized algorithm (ratio of type 1 to type 2 codes ≥ 4, and at least 1 insulin prescription within 90 days) performed highly accurately at ages at diagnosis < 20 years, but it also generated close estimates of the proportion of T1D among adults, as misclassified T1D and T2D cases were approximately balanced. Thus, the optimized algorithm could be applied to diabetes incidence and prevalence studies. Other algorithms may be better suited for cohort studies or other designs. For example, an adult-onset T1D cohort study could use the “high PPV for type 1” algorithm (ratio of type 1 to type 2 codes ≥ 4, and multiple daily injections with no other glucose-lowering medication prescription) to maximize PPV. Alternatively, a case-finding study designed to identify as many people with T1D as possible might apply the “high sensitivity for type 1” algorithm (ratio of type 1 to type 2 codes ≥ 4, or at least 1 insulin prescription within 90 days). A cohort study of T2D among adults could apply the “high PPV for type 2” (equivalent to “high sensitivity for type 1”) algorithm, although all 3 algorithms performed well considering the relatively high T2D prevalence in adults.

Our large register-based validation study is the first to specifically distinguish T1D and T2D in Asians, using routinely available encounter codes and prescriptions in a population-wide EHR within a public universal healthcare context. Unlike previous studies, we demonstrated the critical importance of age at diagnosis, defining separate derivation and validation cohorts to avoid overfitting. However, there are some limitations to note. As in other public healthcare settings, we did not have access to routine autoantibody or C-peptide testing to verify diagnoses of T1D. We could not include the entire HKDSD or externally validate because full chart access was only authorized for the HKDR. However, the HKDR represents a large geographic region of Hong Kong, which has a single publicly administered healthcare system serving its entire population. Although socioeconomic status variables were not captured in our databases, other baseline characteristics were highly similar between the HKDR and HKDSD, supporting the generalizability of our algorithms. Research platforms such as the HA’s Data Collaboration Lab should allow more comprehensive use of EHR data to improve diabetes classification using more complex methodologies and to enhance population research [32–34].

## Conclusions

In summary, we developed and validated a set of algorithms to accurately classify diabetes type for different ages at diagnosis using population-level health data. As EHRs become increasingly available, our approach may be applied to generate similar algorithms in other settings. These algorithms can be applied to future studies to characterize incidence, prevalence, and other statistics separately for T1D and T2D—especially in China and other populations where these statistics have never been measured [11].

## Supplementary information

**Additional file 1.** Demographic characteristics of the New Territories East Cluster (NTEC) population and the overall Hong Kong population [2, 3]

## Data Availability

The data set supporting the conclusions of this article is not publicly available. Interested researchers may apply for access through Shirley Au, for the Secretary of the Central Panel on Administrative Assessment of External Data Requests, Hospital Authority, Hong Kong Special Administrative Region (e-mail, hacpaaedr@ha.org.hk).

## Abbreviations

A1C: Glycated haemoglobin A_1c_
DPP-4: Dipeptidyl peptidase-4
eGFR: Estimated glomerular filtration rate
EHR: Electronic health record
FN: False negative
FP: False positive
GLP-1: Glucagon-like peptide-1
HA: Hong Kong Hospital Authority
HDL-C: High-density lipoprotein cholesterol
HKDR: Hong Kong Diabetes Registry
HKDSD: Hong Kong Diabetes Surveillance Database
ICD-9: International Statistical Classification of Diseases and Related Health Problems version 9
IQR: Interquartile range
LDL-C: Low-density lipoprotein cholesterol
NPV: Negative predictive value
PPV: Positive predictive value
RAS: Renin-angiotensin system
SEARCH: SEARCH for Diabetes in Youth Study
SGLT2: Sodium-glucose transport protein 2
T1D: Type 1 diabetes
T2D: Type 2 diabetes
TN: True negative
TP: True positive
