# Supplementary material for: Development and validation of algorithms to classify type 1 and 2 diabetes according to age at diagnosis using electronic health records
Source: BMC Med Res Methodol. 2020 Feb 24;20:35. doi: 10.1186/s12874-020-00921-3 (PMC7038546; doi:10.1186/s12874-020-00921-3)
Supplement: Supplementary file 1 — Additional file 1. Demographic characteristics of the New Territories East Cluster (NTEC) population and the overall Hong Kong population [2, 3] [file 12874_2020_921_MOESM1_ESM.pdf]

Additional File

I. Additional Methodology

The Hong Kong Diabetes Register comprises people with diabetes living in the "New Territories East Cluster," which is the geographically largest catchment area of the Hong Kong Hospital Authority. The age distribution, income, and education of people in the New Territories East Cluster are highly similar to the overall Hong Kong population [1].

Supplementary Table 1 Demographic characteristics of the New Territories East Cluster (NTEC) population and the overall Hong Kong population [2, 3]

	2006 Census		2016 Census	
	NTEC	Hong Kong	NTEC	Hong Kong
Population (millions)	1.2	6.9	1.3	7.3
Median age (years)	39	39	44	43
Proportion aged ≥65 years (%)	10.0	12.4	15.7	15.8
Median monthly household income ($)*	2334	2213	3300	3205
Post-secondary education (%)†	22.8	23.0	32.1	32.7

*In US dollars, using a conversion rate of 7.8 Hong Kong dollars = 1 US dollar

†Among people aged ≥15 years.

Reproduced with permission from Ke C, Lau E, Shah BR, Stukel TA, Ma RC, So W-Y, et al. Excess Burden of Mental Illness and Hospitalization in Young-Onset Type 2 Diabetes: A Population-Based Cohort Study. Annals of Internal Medicine. 2019;170:145–54. https://annals.org/aim/article-abstract/2720956/excess-burden-mental-illness-hospitalization-young-onset-type-2-diabetes ©American College of Physicians.

Supplementary Table 2 Classification of insulin preparations by duration of action. We only included long-term insulin prescriptions (duration ≥28 days).

Duration of Action	Name of Preparation
Short	Insulin aspart human analog<br>Insulin lispro human analog<br>Insulin neutral
Intermediate	Insulin isophane<br>Insulin zinc susp
Long (basal)	Insulin detemir<br>Insulin glargine
Combination (premixed)	Insulin aspart 30% + aspart prot 70%<br>Human isophane70% Neutral30%<br>Insulin human Mixtard 10HM<br>Insulin human Mixtard 20HM<br>Insulin human Mixtard 40HM<br>Insulin human Mixtard 50HM<br>Insulin isophane 70% + Neutral 30%<br>Insulin lispro (Humalog Mix25)<br>Insulin lispro (Humalog Mix50)

Supplementary Table 3 Glucose-lowering medication prescriptions (excluding insulin) included in the study

Class	Medication Name
Sulfonylureas	Chlopropamide
	Acetohexamide
	Glibenclamide
	Gliclazide
	Glimepiride
	Glipizide
	Tobutamide
Biguanides	Metformin
Other	Acarbose
	Actosmet
	Alogliptin
	Avandamet
	Dapagliflozin
	Empagliflozin
	Exenatide
	Galvusmet
	Janumet
	Kombiglyze
	Linagliptin
	Liraglutide
	Lixisenatide
	Oseni
	Pioglitazone
	Rosiglitazone
	Saxagliptin
	Sitagliptin
	Trajenta Duo
	Vildagliptin

II. Additional Results

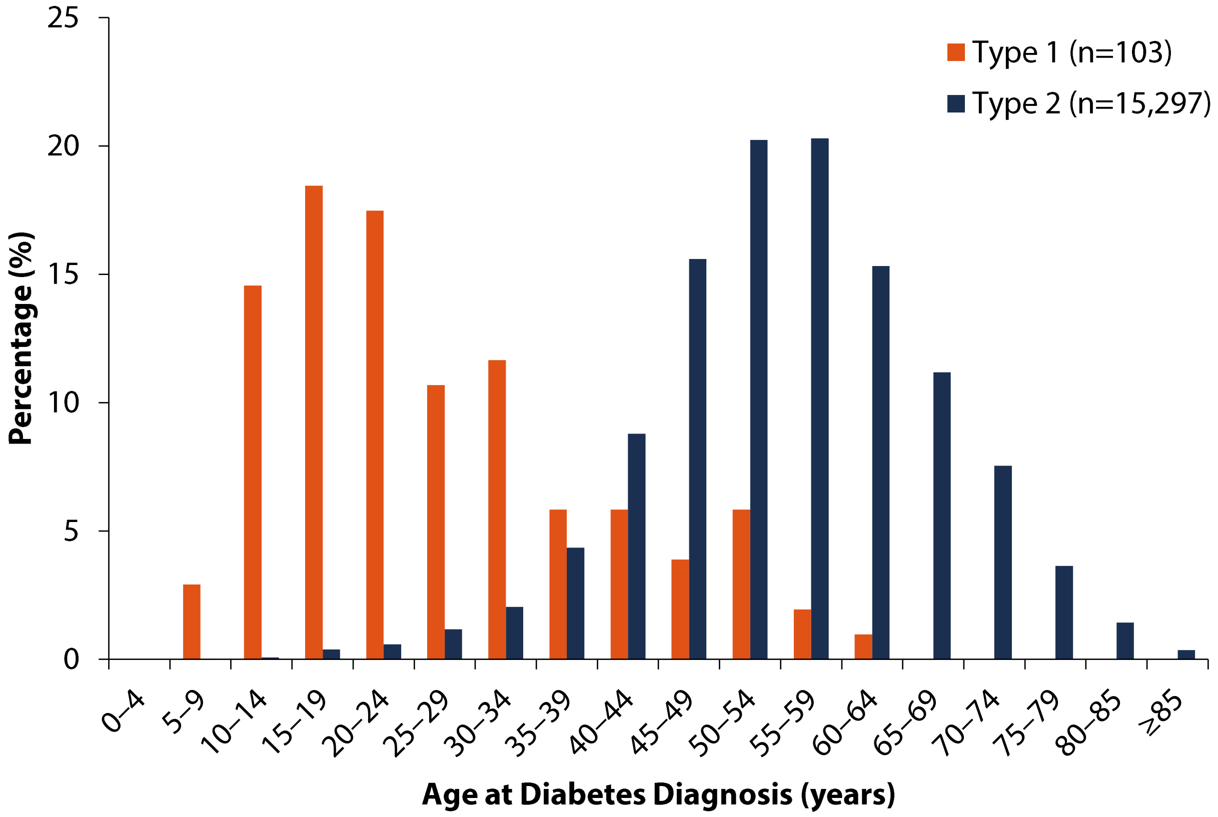

Supplementary Figure 1 Distributions of age at diagnosis (years) of type 1 and 2 diabetes in the Hong Kong Diabetes Register (2002–15). The mean age at diagnosis was 27.0 (standard deviation 13.1) years for type 1 diabetes and 55.9 (11.5) years for type 2 diabetes. The median age at diagnosis was 23.3 (interquartile range 17.4) years for type 1 diabetes and 55.8 (14.8) years for type 2 diabetes.

Supplementary Table 4 Performance of candidate prescription algorithms developed using prescription and renal function (estimated glomerular filtration rate, eGFR) criteria in the derivation cohort. For each candidate algorithm, values in the type 1 and type 2 diabetes columns indicate the number and percentage of individuals satisfying the algorithm (sensitivity). Positive predictive values (PPV) for predicting type 1 are shown in the right column. The algorithms with the highest sensitivity (E) and PPV (F) are indicated. See Table 2 for algorithms A–D (without renal function criteria). Algorithm E had a worse sensitivity than algorithm C, but algorithm F had a better PPV than algorithm D.

Candidate Prescription Algorithms (including renal function criteria)	Type 1 (*n*=60)	Type 2 (*n*=10,136)	PPV
No eGFR values <60 mL/min/1.73 m^2 within 365 days of first insulin prescription	50 (83.3)	293 (2.9)	14.6
and at least 1 insulin prescription within 90 days (E)	50 (83.3)	215 (2.1)	18.9
and at least 1 insulin prescription within 180 days	50 (83.3)	242 (2.4)	17.1
and at least 1 insulin prescription within 365 days	50 (83.3)	272 (2.7)	15.5
and at least 1 insulin prescription with no other glucose-lowering medication prescription	34 (56.7)	20 (0.2)	63.0
and at least 1 insulin prescription with no other glucose-lowering medication prescription within 90 days	38 (63.3)	122 (1.2)	23.8
and at least 1 insulin prescription with no other glucose-lowering medication prescription within 180 days	39 (65.0)	153 (1.5)	20.3
and at least 1 insulin prescription with no other glucose-lowering medication prescription within 365 days	40 (66.7)	1911 (18.9)	2.0
and at least 1 insulin prescription with no other glucose-lowering medication prescription except metformin	45 (75.0)	76 (0.7)	37.2
and at least 1 insulin prescription with no other glucose-lowering medication prescription except metformin within 90 days	47 (78.3)	16 (0.2)	74.6
and at least 1 insulin prescription with no other glucose-lowering medication prescription except metformin within 180 days	48 (80.0)	185 (1.8)	20.6
and at least 1 insulin prescription with no other glucose-lowering medication prescription except metformin within 365 days	48 (80.0)	226 (2.2)	17.5
and multiple daily injections*	41 (68.3)	51 (0.5)	44.6
and multiple daily injections within 90 days	7 (11.7)	4 (0.0)	63.6
and multiple daily injections within 180 days	8 (13.3)	8 (0.1)	50.0
and multiple daily injections within 365 days	12 (20.0)	11 (0.1)	52.2
and multiple daily injections with no other glucose-lowering medication prescription (F)	30 (50.0)	7 (0.1)	81.1
and multiple daily injections with no other glucose-lowering medication prescription within 90 days	7 (11.7)	2 (0.0)	77.8
and multiple daily injections with no other glucose-lowering medication prescription within 180 days	8 (13.3)	6 (0.1)	57.1
and multiple daily injections with no other glucose-lowering medication prescription within 365 days	12 (20.0)	10 (0.1)	54.6
and multiple daily injections with no other glucose-lowering medication prescription except metformin	39 (65.0)	16 (0.2)	70.9
and multiple daily injections with no other glucose-lowering medication prescription except metformin within 90 days	7 (11.7)	2 (0.0)	77.8
and multiple daily injections with no other glucose-lowering medication prescription except metformin within 180 days	8 (13.3)	6 (0.1)	57.1
and multiple daily injections with no non-insulin prescription except metformin within 365 days	12 (20.0)	10 (0.1)	54.6

Abbreviations: PPV, positive predictive value; eGFR, estimated glomerular filtration rate
*Multiple daily injections defined as prescriptions for long-acting and short-acting insulin initiated within the same month
All indicated durations are counted from the diagnosis date. If no duration is indicated, all available data were utilized (up to 2016).

Supplementary Table 5 Test characteristics of single (A–C, F) and combination algorithms (including renal function criteria) for classifying type 1 diabetes compared to the reference standard in the derivation cohort, stratified by age at diagnosis. Algorithm D was substituted with algorithm F because the latter had a higher positive predictive value (PPV; Supplementary Table 4); algorithms A–C remain unchanged from the original analysis because they did not require modification on the basis of renal function (A, B) or the sensitivity was not improved by the inclusion of renal function criteria (C; see Supplementary Table 4). Sensitivity, specificity, positive predictive value (PPV) and negative predictive value (NPV) are percentages with 95% confidence intervals. Cohen's kappa coefficient represents agreement after agreement due to chance is removed (1.0 indicates perfect agreement). The "Type 1 Proportion" columns refer to the percentage of people in the cohort with diabetes classified as having type 1 diabetes using each algorithm ("Calculated") and the reference standard ("True"). The best overall algorithms are marked (*=highest sensitivity, †=highest PPV, ‡=highest kappa coefficient).

Algorithm	TP	FP	FN	TN	Sensitivity	Specificity	PPV	NPV	Kappa	Type 1 Proportion (%)	
										Calculated	True
**All Ages**											
Ratio of type 1 to type 2 codes ≥0.5 (A)	50	97	10	10039	83.3 (71.5, 91.7)	99.0 (98.8, 99.2)	34.0 (26.4, 42.3)	99.9 (99.8, 100.0)	0.48	1.4	
Ratio of type 1 to type 2 codes ≥4 (B)	43	32	17	10104	71.7 (58.6, 82.5)	99.7 (99.6, 99.8)	57.3 (45.4, 68.7)	99.8 (99.7, 99.9)	0.63	0.7	
At least 1 insulin prescription within 90 days (C)	58	615	2	9521	96.7 (88.5, 99.6)	93.9 (93.5, 94.4)	8.6 (6.6, 11.0)	100.0 (99.9, 100.0)	0.15	6.6	
Normal eGFR and multiple daily injections with no other glucose-lowering medication prescription (F)	30	7	30	10129	50.0 (36.8, 63.2)	99.9 (99.9, 100.0)	81.1 (64.8, 92.0)	99.7 (99.6, 99.8)	0.62	0.4	
A and C	49	52	11	10084	81.7 (69.6, 90.5)	99.5 (99.3, 99.6)	48.5 (38.4, 58.7)	99.9 (99.8, 99.9)	0.61	1.0	0.6
A and F	26	4	34	10132	43.3 (30.6, 56.8)	100.0 (99.9, 100.0)	86.7 (69.3, 96.2)	99.7 (99.5, 99.8)	0.58	0.3	
B and C‡	42	19	18	10117	70.0 (56.8, 81.2)	99.8 (99.7, 99.9)	68.9 (55.7, 80.1)	99.8 (99.7, 99.9)	0.69	0.6	
B and F†	23	3	37	10133	38.3 (26.1, 51.8)	100.0 (99.9, 100.0)	88.5 (69.8, 97.6)	99.6 (99.5, 99.7)	0.53	0.3	
A or C	59	660	1	9476	98.3 (91.1, 100.0)	93.5 (93.0, 94.0)	8.2 (6.3, 10.5)	100.0 (99.9, 100.0)	0.14	7.1	
A or F	54	100	6	10036	90.0 (79.5, 96.2)	99.0 (98.8, 99.2)	35.1 (27.6, 43.2)	99.9 (99.9, 100.0)	0.50	1.5	
B or C*	59	628	1	9508	98.3 (91.1, 100.0)	93.8 (93.3, 94.3)	8.6 (6.6, 10.9)	100.0 (99.9, 100.0)	0.15	6.7	
B or F	50	36	10	10100	83.3 (71.5, 91.7)	99.6 (99.5, 99.8)	58.1 (47.0, 68.7)	99.9 (99.8, 100.0)	0.68	0.8	
Age <20 years											
Ratio of type 1 to type 2 codes ≥0.5 (A)	23	9	0	26	100.0 (85.2, 100.0)	74.3 (56.7, 87.5)	71.9 (53.3, 86.3)	100.0 (86.8, 100.0)	0.70	55.2	
Ratio of type 1 to type 2 codes ≥4 (B)	21	5	2	30	91.3 (72.0, 98.9)	85.7 (69.7, 95.2)	80.8 (60.6, 93.4)	93.8 (79.2, 99.2)	0.75	44.8	
At least 1 insulin prescription within 90 days (C)	23	13	0	22	100.0 (85.2, 100.0)	62.9 (44.9, 78.5)	63.9 (46.2, 79.2)	100.0 (84.6, 100.0)	0.57	62.1	
Normal eGFR and multiple daily injections with no other glucose-lowering medication prescription (F)	15	2	8	33	65.2 (42.7, 83.6)	94.3 (80.8, 99.3)	88.2 (63.6, 98.5)	80.5 (65.1, 91.2)	0.62	29.3	39.7
A and C	23	8	0	27	100.0 (85.2, 100.0)	77.1 (59.9, 89.6)	74.2 (55.4, 88.1)	100.0 (87.2, 100.0)	0.73	53.4	
A and F	15	2	8	33	65.2 (42.7, 83.6)	94.3 (80.8, 99.3)	88.2 (63.6, 98.5)	80.5 (65.1, 91.2)	0.62	29.3	
B and C	21	5	2	30	91.3 (72.0, 98.9)	85.7 (69.7, 95.2)	80.8 (60.6, 93.4)	93.8 (79.2, 99.2)	0.75	44.8	

Supplementary Table 5 Test characteristics of single (A–C, F) and combination algorithms (including renal function criteria) for classifying type 1 diabetes compared to the reference standard in the derivation cohort, stratified by age at diagnosis *(Continued)*

Algorithm	TP	FP	FN	TN	Sensitivity	Specificity	PPV	NPV	Kappa	Type 1 Proportion (%)	
										Calculated	True
B and F	14	2	9	33	60.9 (38.5, 80.3)	94.3 (80.8, 99.3)	87.5 (61.7, 98.4)	78.6 (63.2, 89.7)	0.58	27.6	
A or C	23	14	0	21	100.0 (85.2, 100.0)	60.0 (42.1, 76.1)	62.2 (44.8, 77.5)	100.0 (83.9, 100.0)	0.54	63.8	
A or F	23	9	0	26	100.0 (85.2, 100.0)	74.3 (56.7, 87.5)	71.9 (53.3, 86.3)	100.0 (86.8, 100.0)	0.70	55.2	39.7
B or C	23	13	0	22	100.0 (85.2, 100.0)	62.9 (44.9, 78.5)	63.9 (46.2, 79.2)	100.0 (84.6, 100.0)	0.57	62.1	
B or F	22	5	1	30	95.7 (78.1, 99.9)	85.7 (69.7, 95.2)	81.5 (61.9, 93.7)	96.8 (83.3, 99.9)	0.79	46.6	
**Age 20–39 years**											
Ratio of type 1 to type 2 codes ≥0.5 (A)	21	53	5	700	80.8 (60.6, 93.4)	93.0 (90.9, 94.7)	28.4 (18.5, 40.1)	99.3 (98.4, 99.8)	0.39	9.5	
Ratio of type 1 to type 2 codes ≥4 (B)	19	16	7	737	73.1 (52.2, 88.4)	97.9 (96.6, 98.8)	54.3 (36.6, 71.2)	99.1 (98.1, 99.6)	0.61	4.5	
At least 1 insulin prescription within 90 days (C)	25	80	1	673	96.2 (80.4, 99.9)	89.4 (87.0, 91.5)	23.8 (16.0, 33.1)	99.9 (99.2, 100.0)	0.35	13.5	
Normal eGFR and multiple daily injections with no other glucose-lowering medication prescription (F)	12	2	14	751	46.2 (26.6, 66.6)	99.7 (99.0, 100.0)	85.7 (57.2, 98.2)	98.2 (96.9, 99.0)	0.59	1.8	
A and C	20	23	6	730	76.9 (56.4, 91.0)	96.9 (95.5, 98.1)	46.5 (31.2, 62.3)	99.2 (98.2, 99.7)	0.56	5.5	3.3
A and F	9	0	17	753	34.6 (17.2, 55.7)	100.0 (99.5, 100.0)	100.0 (66.4, 100.0)	97.8 (96.5, 98.7)	0.51	1.2	
B and C	18	8	8	745	69.2 (48.2, 85.7)	98.9 (97.9, 99.5)	69.2 (48.2, 85.7)	98.9 (97.9, 99.5)	0.68	3.3	
B and F	9	0	17	753	34.6 (17.2, 55.7)	100.0 (99.5, 100.0)	100.0 (66.4, 100.0)	97.8 (96.5, 98.7)	0.51	1.2	
A or C	26	110	0	643	100.0 (86.8, 100.0)	85.4 (82.7, 87.8)	19.1 (12.9, 26.7)	100.0 (99.4, 100.0)	0.28	17.5	
A or F	24	55	2	698	92.3 (74.9, 99.1)	92.7 (90.6, 94.5)	30.4 (20.5, 41.8)	99.7 (99.0, 100.0)	0.43	10.1	
B or C	26	88	0	665	100.0 (86.8, 100.0)	88.3 (85.8, 90.5)	22.8 (15.5, 31.6)	100.0 (99.4, 100.0)	0.34	14.6	
B or F	22	18	4	735	84.6 (65.1, 95.6)	97.6 (96.2, 98.6)	55.0 (38.5, 70.7)	99.5 (98.6, 99.9)	0.65	5.1	
**Age ≥40 years**											
Ratio of type 1 to type 2 codes ≥0.5 (A)	6	35	5	9313	54.5 (23.4, 83.3)	99.6 (99.5, 99.7)	14.6 (5.6, 29.2)	99.9 (99.9, 100.0)	0.23	0.4	
Ratio of type 1 to type 2 codes ≥4 (B)	3	11	8	9337	27.3 (6.0, 61.0)	99.9 (99.8, 99.9)	21.4 (4.7, 50.8)	99.9 (99.8, 100.0)	0.24	0.1	
At least 1 insulin prescription within 90 days (C)	10	522	1	8826	90.9 (58.7, 99.8)	94.4 (93.9, 94.9)	1.9 (0.9, 3.4)	100.0 (99.9, 100.0)	0.03	5.7	
Normal eGFR and multiple daily injections with no other glucose-lowering medication prescription (F)	3	3	8	9345	27.3 (6.0, 61.0)	100.0 (99.9, 100.0)	50.0 (11.8, 88.2)	99.9 (99.8, 100.0)	0.35	0.1	
A and C	6	21	5	9327	54.5 (23.4, 83.3)	99.8 (99.7, 99.9)	22.2 (8.6, 42.3)	99.9 (99.9, 100.0)	0.31	0.3	0.1
A and F	2	2	9	9346	18.2 (2.3, 51.8)	100.0 (99.9, 100.0)	50.0 (6.8, 93.2)	99.9 (99.8, 100.0)	0.27	0.0	
B and C	3	6	8	9342	27.3 (6.0, 61.0)	99.9 (99.9, 100.0)	33.3 (7.5, 70.1)	99.9 (99.8, 100.0)	0.30	0.1	
B and F	0	1	11	9347	100.0 (71.5, 100.0)	100.0 (99.9, 100.0)	100.0 (2.5, 100.0)	99.9 (99.8, 99.9)	0.00	0.0	
A or C	10	536	1	8812	90.9 (58.7, 99.8)	94.3 (93.8, 94.7)	1.8 (0.9, 3.3)	100.0 (99.9, 100.0)	0.03	5.8	
A or F	7	36	4	9312	63.6 (30.8, 89.1)	99.6 (99.5, 99.7)	16.3 (6.8, 30.7)	100.0 (99.9, 100.0)	0.26	0.5	
B or C	10	527	1	8821	90.9 (58.7, 99.8)	94.4 (93.9, 94.8)	1.9 (0.9, 3.4)	100.0 (99.9, 100.0)	0.03	5.7	
B or F	6	13	5	9335	54.5 (23.4, 83.3)	99.9 (99.8, 99.9)	31.6 (12.6, 56.6)	99.9 (99.9, 100.0)	0.40	0.2	

Abbreviations: TP, true positive; FP, false positive; FN, false negative; TN, true negative; PPV, positive predictive value; NPV, negative predictive value; eGFR, estimated glomerular filtration rate (normal eGFR defined as having no eGFR values <60 mL/min/1.73 m^2 within 365 days of first insulin prescription)
Multiple daily injections was defined as prescriptions for long-acting and short-acting insulin initiated within the same month

Supplementary Table 6 Test characteristics of the high sensitivity, high positive predictive value (PPV, including renal function criterion), and balanced algorithms for classifying type 1 diabetes compared to the reference standard in the validation cohort, stratified by age at diagnosis. Sensitivity, specificity, PPV and negative predictive value (NPV) are percentages with 95% confidence intervals. Cohen's kappa coefficient represents agreement after agreement due to chance is removed (1.0 indicates perfect agreement). The "Type 1 Proportion" columns refer to the percentage of people in the cohort with diabetes classified as type 1 using each algorithm ("Calculated") and the reference standard ("True").

Algorithm	TP	FP	FN	TN	Sensitivity	Specificity	PPV	NPV	Kappa	Type 1 Proportion (%)	
										Calculated	True
High Sensitivity for Type 1: ratio of type 1 to type 2 codes ≥4, or at least 1 insulin prescription within 90 days											
All Ages	41	280	2	4778	95.3 (84.2, 99.4)	94.5 (93.8, 95.1)	12.8 (9.3, 16.9)	100.0 (99.8, 100.0)	0.21	6.3	0.8
Age <20 years	14	6	0	21	100.0 (76.8, 100.0)	77.8 (57.7, 91.4)	70.0 (45.7, 88.1)	100.0 (83.9, 100.0)	0.71	48.8	34.1
Age 20–39 years	19	41	2	300	90.5 (69.6, 98.8)	88.0 (84.0, 91.2)	31.7 (20.3, 45.0)	99.3 (97.6, 99.9)	0.42	16.6	5.8
Age ≥40 years	8	233	0	4457	100.0 (63.1, 100.0)	95.0 (94.4, 95.6)	3.3 (1.4, 6.4)	100.0 (99.9, 100.0)	0.06	5.1	0.2
High PPV for Type 1: ratio of type 1 to type 2 codes ≥4 and normal eGFR and multiple daily injections* with no glucose-lowering medication prescription excluding insulin											
All Ages	12	0	31	5058	27.9 (15.3, 43.7)	100.0 (99.9, 100.0)	100.0 (73.5, 100.0)	99.4 (99.1, 99.6)	0.43	0.2	0.8
Age <20 years	8	0	6	27	57.1 (28.9, 82.3)	100.0 (87.2, 100.0)	100.0 (63.1, 100.0)	81.8 (64.5, 93.0)	0.64	19.5	34.1
Age 20–39 years	4	0	17	341	19.0 (5.4, 41.9)	100.0 (98.9, 100.0)	100.0 (39.8, 100.0)	95.3 (92.5, 97.2)	0.31	1.1	5.8
Age ≥40 years	0	0	8	4690	0.0 (0, 36.9)	100.0 (99.9, 100.0)	Undefined	99.8 (99.7, 99.9)	0.00	0.0	0.2
Balanced: ratio of type 1 to type 2 codes ≥4, and at least 1 insulin prescription within 90 days											
All Ages	28	9	15	5049	65.1 (49.1, 79.0)	99.8 (99.7, 99.9)	75.7 (58.8, 88.2)	99.7 (99.5, 99.8)	0.70	0.7	0.8
Age <20 years	12	0	2	27	85.7 (57.2, 98.2)	100.0 (87.2, 100.0)	100.0 (73.5, 100.0)	93.1 (77.2, 99.2)	0.89	29.3	34.1
Age 20–39 years	12	7	9	334	57.1 (34.0, 78.2)	97.9 (95.8, 99.2)	63.2 (38.4, 83.7)	97.4 (95.1, 98.8)	0.58	5.2	5.8
Age ≥40 years	4	2	4	4688	50.0 (15.7, 84.3)	100.0 (99.8, 100.0)	66.7 (22.3, 95.7)	99.9 (99.8, 100.0)	0.57	0.1	0.2

Abbreviations: TP, true positive; FP, false positive; FN, false negative; TN, true negative; PPV, positive predictive value; NPV, negative predictive value; eGFR, estimated glomerular filtration rate (normal eGFR defined as having no eGFR values <60 mL/min/1.73 m^2 within 365 days of first insulin prescription)

*Multiple daily injections was defined as prescriptions for long-acting and short-acting insulin initiated in the same month

Supplementary Table 7 Test characteristics of selected single and combination algorithms for classifying type 2 diabetes compared to the reference standard in the derivation cohort, stratified by age at diagnosis. Algorithms are worded with respect to type 1 diabetes, so type 2 cases are those that do *not* satisfy the listed criteria. Sensitivity, specificity, positive predictive value (PPV) and negative predictive value (NPV) are percentages with 95% confidence intervals. Cohen's kappa coefficient represents agreement after agreement due to chance is removed (1.0 indicates perfect agreement). The "Type 2 Proportion" columns refer to the percentage of people in the cohort with diabetes classified as having type 2 using each algorithm ("Calculated") and the reference standard ("True"). The best overall algorithms are marked (*=high PPV for type 2 [equivalent to high sensitivity for type 1], †=high sensitivity for type 2 [equivalent to high PPV for type 1], ‡=balanced).

Algorithm	TP	FP	FN	TN	Sensitivity	Specificity	PPV	NPV	Kappa	Type 2 Proportion (%)	
										Calculated	True
**All Ages**											
Ratio of type 1 to type 2 codes ≥0.5 (A)	10,039	10	97	50	99.0 (98.8, 99.2)	83.3 (71.5, 91.7)	99.9 (99.8, 100.0)	34.0 (26.4, 42.3)	0.48	98.6	
Ratio of type 1 to type 2 codes ≥4 (B)	10,104	17	32	43	99.7 (99.6, 99.8)	71.7 (58.6, 82.5)	99.8 (99.7, 99.9)	57.3 (45.4, 68.7)	0.63	99.3	
At least 1 insulin prescription within 90 days (C)	9,521	2	615	58	93.9 (93.5, 94.4)	96.7 (88.5, 99.6)	100.0 (99.9, 100.0)	8.6 (6.6, 11.0)	0.15	93.4	
Multiple daily injections with no other glucose-lowering medication prescription (D)	10,127	28	9	32	99.9 (99.8, 100.0)	53.3 (40.0, 66.3)	99.7 (99.6, 99.8)	78.0 (62.4, 89.4)	0.63	99.6	
A and C	10,084	11	52	49	99.5 (99.3, 99.6)	81.7 (69.6, 90.5)	99.9 (99.8, 99.9)	48.5 (38.4, 58.7)	0.61	99.0	
A and D	10,131	32	5	28	100.0 (99.9, 100.0)	46.7 (33.7, 60.0)	99.7 (99.6, 99.8)	84.8 (68.1, 94.9)	0.60	99.7	98.6
B and C‡	10,117	18	19	42	99.8 (99.7, 99.9)	70.0 (56.8, 81.2)	99.8 (99.7, 99.9)	68.9 (55.7, 80.1)	0.69	99.4	
B and D†	10,132	35	4	25	100.0 (99.9, 100.0)	41.7 (29.1, 55.1)	99.7 (99.5, 99.8)	86.2 (68.3, 96.1)	0.56	99.7	
A or C	9,476	1	660	59	93.5 (93.0, 94.0)	98.3 (91.1, 100.0)	100.0 (99.9, 100.0)	8.2 (6.3, 10.5)	0.14	92.9	
A or D	10,035	6	101	54	99.0 (98.8, 99.2)	90.0 (79.5, 96.2)	99.9 (99.9, 100.0)	34.8 (27.4, 42.9)	0.50	98.5	
B or C*	9,508	1	628	59	93.8 (93.3, 94.3)	98.3 (91.1, 100.0)	100.0 (99.9, 100.0)	8.6 (6.6, 10.9)	0.15	93.3	
B or D	10,099	10	37	50	99.6 (99.5, 99.7)	83.3 (71.5, 91.7)	99.9 (99.8, 100.0)	57.5 (46.4, 68.0)	0.68	99.1	
**Age <20 years**											
Ratio of type 1 to type 2 codes ≥0.5 (A)	26	0	9	23	74.3 (56.7, 87.5)	100.0 (85.2, 100.0)	100.0 (86.8, 100.0)	71.9 (53.3, 86.3)	0.70	44.8	
Ratio of type 1 to type 2 codes ≥4 (B)	30	2	5	21	85.7 (69.7, 95.2)	91.3 (72.0, 98.9)	93.8 (79.2, 99.2)	80.8 (60.6, 93.4)	0.75	55.2	
At least 1 insulin prescription within 90 days (C)	22	0	13	23	62.9 (44.9, 78.5)	100.0 (85.2, 100.0)	100.0 (84.6, 100.0)	63.9 (46.2, 79.2)	0.57	37.9	
Multiple daily injections with no other glucose-lowering medication prescription (D)	32	7	3	16	91.4 (76.9, 98.2)	69.6 (47.1, 86.8)	82.1 (66.5, 92.5)	84.2 (60.4, 96.6)	0.63	67.2	
A and C	27	0	8	23	77.1 (59.9, 89.6)	100.0 (85.2, 100.0)	100.0 (87.2, 100.0)	74.2 (55.4, 88.1)	0.73	46.6	
A and D	33	7	2	16	94.3 (80.8, 99.3)	69.6 (47.1, 86.8)	82.5 (67.2, 92.7)	88.9 (65.3, 98.6)	0.66	69.0	98.6
B and C	30	2	5	21	85.7 (69.7, 95.2)	91.3 (72.0, 98.9)	93.8 (79.2, 99.2)	80.8 (60.6, 93.4)	0.75	55.2	
B and D	33	8	2	15	94.3 (80.8, 99.3)	65.2 (42.7, 83.6)	80.5 (65.1, 91.2)	88.2 (63.6, 98.5)	0.62	70.7	
A or C	21	0	14	23	60.0 (42.1, 76.1)	100.0 (85.2, 100.0)	100.0 (83.9, 100.0)	62.2 (44.8, 77.5)	0.54	36.2	
A or D	25	0	10	23	71.4 (53.7, 85.4)	100.0 (85.2, 100.0)	100.0 (86.3, 100.0)	69.7 (51.3, 84.4)	0.66	43.1	
B or C	22	0	13	23	62.9 (44.9, 78.5)	100.0 (85.2, 100.0)	100.0 (84.6, 100.0)	63.9 (46.2, 79.2)	0.57	37.9	
B or D	29	1	6	22	82.9 (66.4, 93.4)	95.7 (78.1, 99.9)	96.7 (82.8, 99.9)	78.6 (59.0, 91.7)	0.76	51.7	

Supplementary Table 7 Test characteristics of selected single and combination algorithms for classifying type 2 diabetes compared to the reference standard in the derivation cohort, stratified by age at diagnosis *(Continued)*

Algorithm	TP	FP	FN	TN	Sensitivity	Specificity	PPV	NPV	Kappa	Type 2 Proportion (%)	
										Calculated	True
**Age 20–39 years**											
Ratio of type 1 to type 2 codes ≥0.5 (A)	700	5	53	21	93.0 (90.9, 94.7)	80.8 (60.6, 93.4)	99.3 (98.4, 99.8)	28.4 (18.5, 40.1)	0.39	90.5	
Ratio of type 1 to type 2 codes ≥4 (B)	737	7	16	19	97.9 (96.6, 98.8)	73.1 (52.2, 88.4)	99.1 (98.1, 99.6)	54.3 (36.6, 71.2)	0.61	95.5	
At least 1 insulin prescription within 90 days (C)	673	1	80	25	89.4 (87.0, 91.5)	96.2 (80.4, 99.9)	99.9 (99.2, 100.0)	23.8 (16.0, 33.1)	0.35	86.5	
Multiple daily injections with no other glucose-lowering medication prescription (D)	750	13	3	13	99.6 (98.8, 99.9)	50.0 (29.9, 70.1)	98.3 (97.1, 99.1)	81.3 (54.4, 96.0)	0.61	97.9	
A and C	730	6	23	20	96.9 (95.5, 98.1)	76.9 (56.4, 91.0)	99.2 (98.2, 99.7)	46.5 (31.2, 62.3)	0.56	94.5	
A and D	752	16	1	10	99.9 (99.3, 100.0)	38.5 (20.2, 59.4)	97.9 (96.6, 98.8)	90.9 (58.7, 99.8)	0.53	98.6	98.6
B and C	745	8	8	18	98.9 (97.9, 99.5)	69.2 (48.2, 85.7)	98.9 (97.9, 99.5)	69.2 (48.2, 85.7)	0.68	96.7	
B and D	752	16	1	10	99.9 (99.3, 100.0)	38.5 (20.2, 59.4)	97.9 (96.6, 98.8)	90.9 (58.7, 99.8)	0.53	98.6	
A or C	643	0	110	26	85.4 (82.7, 87.8)	100.0 (86.8, 100.0)	100.0 (99.4, 100.0)	19.1 (12.9, 26.7)	0.28	82.5	
A or D	698	2	55	24	92.7 (90.6, 94.5)	92.3 (74.9, 99.1)	99.7 (99.0, 100.0)	30.4 (20.5, 41.8)	0.43	89.9	
B or C	665	0	88	26	88.3 (85.8, 90.5)	100.0 (86.8, 100.0)	100.0 (99.4, 100.0)	22.8 (15.5, 31.6)	0.34	85.4	
B or D	735	4	18	22	97.6 (96.2, 98.6)	84.6 (65.1, 95.6)	99.5 (98.6, 99.9)	55.0 (38.5, 70.7)	0.65	94.9	
**Age ≥40 years**											
Ratio of type 1 to type 2 codes ≥0.5 (A)	9,313	5	35	6	99.6 (99.5, 99.7)	54.5 (23.4, 83.3)	99.9 (99.9, 100.0)	14.6 (5.6, 29.2)	0.23	99.6	
Ratio of type 1 to type 2 codes ≥4 (B)	9,337	8	11	3	99.9 (99.8, 99.9)	27.3 (6.0, 61.0)	99.9 (99.8, 100.0)	21.4 (4.7, 50.8)	0.24	99.9	
At least 1 insulin prescription within 90 days (C)	8,826	1	522	10	94.4 (93.9, 94.9)	90.9 (58.7, 99.8)	100.0 (99.9, 100.0)	1.9 (0.9, 3.4)	0.03	94.3	
Multiple daily injections with no other glucose-lowering medication prescription (D)	9,345	8	3	3	100.0 (99.9, 100.0)	27.3 (6.0, 61.0)	99.9 (99.8, 100.0)	50.0 (11.8, 88.2)	0.35	99.9	
A and C	9,327	5	21	6	99.8 (99.7, 99.9)	54.5 (23.4, 83.3)	99.9 (99.9, 100.0)	22.2 (8.6, 42.3)	0.31	99.7	
A and D	9,346	9	2	2	100.0 (99.9, 100.0)	18.2 (2.3, 51.8)	99.9 (99.8, 100.0)	50.0 (6.8, 93.2)	0.27	100.0	98.6
B and C	9,342	8	6	3	99.9 (99.9, 100.0)	27.3 (6.0, 61.0)	99.9 (99.8, 100.0)	33.3 (7.5, 70.1)	0.30	99.9	
B and D	9,347	11	1	0	100.0 (99.9, 100.0)	0.0 (0.0, 28.5)	99.9 (99.8, 99.9)	Undefined	0.00	100.0	
A or C	8,812	1	536	10	94.3 (93.8, 94.7)	90.9 (58.7, 99.8)	100.0 (99.9, 100.0)	1.8 (0.9, 3.3)	0.03	94.2	
A or D	9,312	4	36	7	99.6 (99.5, 99.7)	63.6 (30.8, 89.1)	100.0 (99.9, 100.0)	16.3 (6.8, 30.7)	0.26	99.5	
B or C	8,821	1	527	10	94.4 (93.9, 94.8)	90.9 (58.7, 99.8)	100.0 (99.9, 100.0)	1.9 (0.9, 3.4)	0.03	94.3	
B or D	9,335	5	13	6	99.9 (99.8, 99.9)	54.5 (23.4, 83.3)	99.9 (99.9, 100.0)	31.6 (12.6, 56.6)	0.40	99.8	

Abbreviations: TP, true positive; FP, false positive; FN, false negative; TN, true negative; PPV, positive predictive value; NPV, negative predictive value

Multiple daily injections was defined as prescriptions for long-acting and short-acting insulin initiated in the same month

If there were no true negative cases identified, the negative predictive was indicated as “undefined.”

Supplementary Table 8 Test characteristics of the high sensitivity, high positive predictive value (PPV), and balanced algorithms for classifying type 2 diabetes compared to the reference standard in the validation cohort, stratified by age at diagnosis. Algorithms are worded with respect to type 1 diabetes, so type 2 cases are those that do *not* satisfy the listed criteria. Sensitivity, specificity, PPV and negative predictive value (NPV) are percentages with 95% confidence intervals. Cohen's kappa coefficient represents agreement after agreement due to chance is removed (1.0 indicates perfect agreement). The "Type 2 Proportion" columns refer to the percentage of people in the cohort with diabetes classified as type 2 diabetes using each algorithm ("Calculated") and the reference standard ("True").

Algorithm	TP	FP	FN	TN	Sensitivity	Specificity	PPV	NPV	Kappa	Type 2 Proportion (%)	
										Calculated	True
High PPV for Type 2 (high sensitivity for type 1): ratio of type 1 to type 2 codes <4, or at least 1 insulin prescription within 90 days											
All Ages	4778	2	280	41	94.5 (93.8, 95.1)	95.3 (84.2, 99.4)	100.0 (99.8, 100.0)	12.8 (9.3, 16.9)	0.21	93.7	99.2
Age <20 years	21	0	6	14	77.8 (57.7, 91.4)	100.0 (76.8, 100.0)	100.0 (83.9, 100.0)	70.0 (45.7, 88.1)	0.71	51.2	65.9
Age 20–39 years	300	2	41	19	88.0 (84.0, 91.2)	90.5 (69.6, 98.8)	99.3 (97.6, 99.9)	31.7 (20.3, 45.0)	0.42	83.4	94.2
Age ≥40 years	4457	0	233	8	95.0 (94.4, 95.6)	100.0 (63.1, 100.0)	100.0 (99.9, 100.0)	3.3 (1.4, 6.4)	0.06	94.9	99.8
High Sensitivity for Type 2 (high PPV for type 1): ratio of type 1 to type 2 codes ≥4, and multiple daily injections* with no glucose-lowering medication prescription excluding insulin											
All Ages	5058	27	0	16	100.0 (99.9, 100.0)	37.2 (23.0, 53.3)	99.5 (99.2, 99.6)	100.0 (79.4, 100.0)	0.54	99.7	99.2
Age <20 years	27	6	0	8	100.0 (87.2, 100.0)	57.1 (28.9, 82.3)	81.8 (64.5, 93.0)	100.0 (63.1, 100.0)	0.64	80.5	65.9
Age 20–39 years	341	15	0	6	100.0 (98.9, 100.0)	28.6 (11.3, 52.2)	95.8 (93.1, 97.6)	100.0 (54.1, 100.0)	0.43	98.3	94.2
Age ≥40 years	4690	6	0	2	100.0 (99.9, 100.0)	25.0 (3.2, 65.1)	99.9 (99.7, 100.0)	100.0 (15.8, 100.0)	0.40	100	99.8
Balanced: ratio of type 1 to type 2 codes ≥4, and at least 1 insulin prescription within 90 days											
All Ages	5049	15	9	28	99.8 (99.7, 99.9)	65.1 (49.1, 79.0)	99.7 (99.5, 99.8)	75.7 (58.8, 88.2)	0.70	99.3	99.2
Age <20 years	27	2	0	12	100.0 (87.2, 100.0)	85.7 (57.2, 98.2)	93.1 (77.2, 99.2)	100.0 (73.5, 100.0)	0.89	70.7	65.9
Age 20–39 years	334	9	7	12	97.9 (95.8, 99.2)	57.1 (34.0, 78.2)	97.4 (95.1, 98.8)	63.2 (38.4, 83.7)	0.58	94.8	94.2
Age ≥40 years	4688	4	2	4	100.0 (99.8, 100.0)	50.0 (15.7, 84.3)	99.9 (99.8, 100.0)	66.7 (22.3, 95.7)	0.57	99.9	99.8

Abbreviations: TP, true positive; FP, false positive; FN, false negative; TN, true negative; PPV, positive predictive value; NPV, negative predictive value

*Multiple daily injections: defined as prescriptions for long-acting and short-acting insulin initiated in the same month

Supplementary Table 9 Test characteristics of previously published algorithms for predicting type 1 diabetes in the derivation and validation cohorts. Results are displayed according to age at diagnosis. Sensitivity and positive predictive value are percentages with 95% confidence intervals. Age ranges were selected to match the original publication. See footnote for algorithm descriptions.

Algorithm	Age at Diagnosis (years)	Derivation Cohort			Validation Cohort		
		Sensitivity	Positive Predictive Value	Kappa	Sensitivity	Positive Predictive Value	Kappa
Vanderloo (C)*	<20	100.0 (85.2, 100.0)	56.1 (39.7, 71.5)	0.43	100.0 (76.8, 100.0)	70.0 (45.7, 88.1)	0.71
Vanderloo (D)*	<20	100.0 (85.2, 100.0)	56.1 (39.7, 71.5)	0.43	100.0 (76.8, 100.0)	70.0 (45.7, 88.1)	0.71
Klompas	All ages	83.3 (71.5, 91.7)	37.0 (28.9, 45.8)	0.51	86.0 (72.1, 94.7)	50.7 (38.7, 62.6)	0.63
	<20	100.0 (85.2, 100.0)	71.9 (53.3, 86.3)	0.70	100.0 (76.8, 100.0)	93.3 (68.1, 99.8)	0.95
	20–39	80.8 (60.6, 93.4)	31.8 (20.9, 44.4)	0.43	85.7 (63.7, 97.0)	46.2 (30.1, 62.8)	0.57
	≥40	54.5 (23.4, 83.3)	16.2 (6.2, 32.0)	0.25	62.5 (24.5, 91.5)	26.3 (9.1, 51.2)	0.37
Lawrence	<20	100.0 (85.2, 100.0)	71.9 (53.3, 86.3)	0.70	100.0 (76.8, 100.0)	87.5 (61.7, 98.4)	0.90
Zhong (2014)	<20	95.7 (78.1, 99.9)	78.6 (59.0, 91.7)	0.76	85.7 (57.2, 98.2)	100.0 (73.5, 100.0)	0.89
Zhong (2016)	<20	95.7 (78.1, 99.9)	75.9 (56.5, 89.7)	0.72	85.7 (57.2, 98.2)	100.0 (73.5, 100.0)	0.89
Sharma	All ages	50.0 (36.8, 63.2)	32.3 (22.9, 42.7)	0.39	39.5 (25.0, 55.6)	40.5 (25.6, 56.7)	0.39
	<20	73.9 (51.6, 89.8)	85.0 (62.1, 96.8)	0.67	64.3 (35.1, 87.2)	100.0 (66.4, 100.0)	0.70
	20–39	50.0 (29.9, 70.1)	76.5 (50.1, 93.2)	0.59	28.6 (11.3, 52.2)	54.5 (23.4, 83.3)	0.35
	≥40	0.0 (0.0, 28.5)	Undefined	0.00	25.0 (3.2, 65.1)	9.1 (1.1, 29.2)	0.13
Luk	All ages	83.3 (71.5, 91.7)	57.5 (46.4, 68.0)	0.68	76.7 (61.4, 88.2)	63.5 (49.0, 76.4)	0.69
	<20	95.7 (78.1, 99.9)	78.6 (59.0, 91.7)	0.76	100.0 (76.8, 100.0)	100.0 (76.8, 100.0)	1.00
	20–39	84.6 (65.1, 95.6)	55.0 (38.5, 70.7)	0.65	71.4 (47.8, 88.7)	62.5 (40.6, 81.2)	0.64
	≥40	54.5 (23.4, 83.3)	31.6 (12.6, 56.6)	0.40	50.0 (15.5, 84.3)	28.6 (8.4, 58.1)	0.36

*Vanderloo et al. [4]: Algorithm C classified as type 1 if age at diagnosis <10 years, or prescription for insulin with or without metformin within 730 days of diagnosis (we removed the test strip prescriptions criterion due to lack of data); all other cases classified automatically as type 2. Algorithm D defined as type 1 if age at diagnosis <10 years, or prescription for insulin only (presumably without metformin) within 730 days (we removed the test strip prescriptions criterion due to lack of data); all other cases classified automatically as type 2. Algorithms A and B were excluded because they are similar to algorithms C and D but additionally required “Status Indian”

registration, which did not apply to our population. The publication did not specify glucose-lowering medications other than metformin and insulin, so we ignored all other prescriptions.

Klompas et al. [5]: classified as type 1 if ratio type 1 to type 2 diabetes codes >0.5 (all codes included) and no glucose-lowering medication other than metformin; all other cases classified automatically as type 2. Rules 1 and 3–5 were excluded due to lack of data.

Lawrence et al. [6]: classified as type 1 if ≥1 type 1 code; all other cases classified automatically as type 2. We modified this rule to include both outpatient encounter codes and inpatient encounter codes (secondary diagnoses from hospital discharge abstracts) because these codes were indistinguishable in our dataset.

Zhong et al. (2014) [7]: classified as type 1 if ratio of type 1 to total (type 1 and type 2) codes ≥0.5; classified as type 2 if ratio of type 2 to total (type 1 and type 2) codes ≥0.4. We modified these rules to include all encounter codes, rather than billing codes, which do not exist in our setting.

Zhong et al. (2016) [8]: classified as type 1 if ≥1 diabetes code, and ratio of type 1 to total (type 1 and type 2) codes ≥0.6. Other cases were not automatically classified as type 2 but required chart review; therefore we only reported statistics for type 1 diabetes classification. We modified this rule to include all encounter codes, rather than billing codes, which do not exist in our setting.

Sharma et al. [9]: classified as type 1 if ≥1 type 1 code only (i.e., no type 2 codes) and ≥1 prescription for insulin only (i.e., no other glucose-lowering medications), or ≥1 type 1 code only (i.e., no type 2 codes) and ≥1 prescription for insulin and <180 days cumulative duration of all prescriptions for glucose-lowering medications excluding insulin, or any diagnostic code for diabetes (i.e., excluding type 1, as those cases were already included in the preceding criteria) with ≥1 prescription for insulin only (we removed the requirement for "incident cases… or diagnosed… at <35 years of age" because all cases in our database were incident). Classified as type 2 if diagnostic code for type 2 only (i.e., no type 1 codes) and ≥1 prescription for glucose-lowering medications aside from insulin, or any type 1 or type 2 or unspecified diabetes code and prescription for ≥180 days of glucose-lowering medications excluding insulin (with or without insulin prescription), or any type 1 or type 2 or unspecified code and ≥1 prescription of glucose-lowering medications excluding insulin and no prescription for insulin, or type 2 or unspecified diabetes code (i.e., no type 1 codes) with no insulin or other glucose-lowering medication prescriptions. These rules were specified according to our best interpretation of the originally published criteria. Cases that did not meet these definitions were not definitely classified as type 1 or type 2 diabetes. We excluded the criteria that classified diabetes cases "with a degree of uncertainty." In accordance with the study authors, prescriptions without an indicated duration were assigned a duration of 28 days.

Schroeder et al. [10]: algorithm excluded because it was identical to the Klompas et al. [5]

Luk et al. [11]: classified as type 1 if ratio of type 1 to type 2 codes ≥4 or multiple daily injections (defined as prescriptions for long-acting and short-acting insulin initiated in the same month) with no glucose-lowering medication prescription excluding insulin (we applied this algorithm to a study that was published before the present validation study).

If there were no true positive cases identified, the positive predictive was indicated as "undefined."

Supplementary Table 10 Test characteristics of previously published algorithms for predicting type 2 diabetes in the derivation and validation cohorts. Results are displayed according to age at diagnosis (ranges selected to match the original publication). Sensitivity and positive predictive value are percentages with 95% confidence intervals. See footnote for algorithm descriptions.

Algorithm	Age at Diagnosis (years)	Derivation Cohort			Validation Cohort		
		Sensitivity	Positive Predictive Value	Kappa	Sensitivity	Positive Predictive Value	Kappa
Vanderloo (C)*	<20	48.6 (31.4, 66.0)	100.0 (80.5, 100.0)	0.43	77.8 (57.7, 91.4)	100.0 (83.9, 100.0)	0.71
Vanderloo (D)*	<20	48.6 (31.4, 66.0)	100.0 (80.5, 100.0)	0.43	77.8 (57.7, 91.4)	100.0 (83.9, 100.0)	0.71
Klompas	All ages	99.2 (99.0, 99.3)	99.9 (99.8, 100.0)	0.51	99.3 (99.0, 99.5)	99.9 (99.7, 100.0)	0.63
	<20	74.3 (56.7, 87.5)	100.0 (86.8, 100.0)	0.70	96.3 (81.0, 99.9)	100.0 (86.8, 100.0)	0.95
	20–39	94.0 (92.1, 95.6)	99.3 (98.4, 99.8)	0.43	93.8 (90.7, 96.1)	99.1 (97.3, 99.8)	0.57
	≥40	99.7 (99.5, 99.8)	99.9 (99.9, 100.0)	0.25	99.7 (99.5, 99.8)	99.9 (99.8, 100.0)	0.37
Lawrence	<20	74.3 (56.7, 87.5)	100.0 (86.8, 100.0)	0.70	92.6 (75.7, 99.1)	100.0 (86.3, 100.0)	0.90
Zhong (2014)	<20	95.7 (78.1, 99.9)	75.9 (56.5, 89.7)	0.72	85.7 (57.2, 98.2)	75.0 (47.6, 92.7)	0.69
Sharma	All ages	65.0 (51.6, 76.9)	1.2 (0.9, 1.7)	0.01	58.1 (42.1, 73.0)	1.5 (1.0, 2.2)	0.01
	<20	82.6 (61.2, 95.0)	82.6 (61.2, 95.0)	0.71	78.6 (49.2, 95.3)	73.3 (44.9, 92.2)	0.63
	20–39	61.5 (40.6, 79.8)	7.1 (4.1, 11.3)	0.07	52.4 (29.8, 74.3)	12.8 (6.6, 21.7)	0.12
	≥40	36.4 (10.9, 69.2)	0.1 (0.0, 0.4)	0.00	37.5 (8.5, 75.5)	0.2 (0.0, 0.6)	0.00
Ke	All ages	98.6 (98.4, 98.8)	99.9 (99.8, 100.0)	0.39	98.6 (98.3, 98.9)	99.9 (99.7, 100.0)	0.49
	<20	74.3 (56.7, 87.5)	100.0 (86.8, 100.0)	0.70	92.6 (75.7, 99.1)	100.0 (86.3, 100.0)	0.90
	20–39	90.6 (88.3, 92.6)	99.3 (98.3, 99.8)	0.32	91.8 (88.4, 94.5)	99.1 (97.3, 99.8)	0.50
	≥40	99.3 (99.2, 99.5)	99.9 (99.9, 100.0)	0.15	99.2 (98.9, 99.4)	99.9 (99.8, 100.0)	0.19
Luk	All ages	99.6 (99.5, 99.7)	99.9 (99.8, 100.0)	0.68	99.6 (99.4, 99.8)	99.8 (99.6, 99.9)	0.69
	<20	82.9 (66.4, 93.4)	96.7 (82.8, 99.9)	0.76	100.0 (87.2, 100.0)	100.0 (87.2, 100.0)	1.00
	20–39	97.6 (96.2, 98.6)	99.5 (98.6, 99.9)	0.65	97.4 (95.0, 98.8)	98.2 (96.2, 99.3)	0.64
	≥40	99.9 (99.8, 99.9)	99.9 (99.9, 100.0)	0.40	99.8 (99.6, 99.9)	99.9 (99.8, 1000)	0.36

Ke et al. [1]: classified as type 2 if no type 1 diabetes encounter codes (we applied this algorithm to a study that was published before the present validation study).

See Supplementary Table 9 footnote for descriptions of the other algorithms.

References

1. Ke C, Lau E, Shah BR, Stukel TA, Ma RC, So W-Y, et al. Excess Burden of Mental Illness and Hospitalization in Young-Onset Type 2 Diabetes: A Population-Based Cohort Study. Ann Intern Med. 2019;170:145–54.

2. Hospital Authority. Helping People Stay Healthy: Strategic Service Plan 2009-2012. Strategic Service Plan. Hong Kong: Hospital Authority; 2009. http://www.ha.org.hk/ho/corpcomm/Strategic%20Plan/2009-12.pdf. Accessed 31 January 2020.

3. Census and Statistics Department, Hong Kong Special Administrative Region. 2016 Population By-census. 2016 Population By-Census. 2016. https://www.bycensus2016.gov.hk/en/. Accessed 31 January 2020.

4. Vanderloo SE, Johnson JA, Reimer K, McCrea P, Nuernberger K, Krueger H, et al. Validation of classification algorithms for childhood diabetes identified from administrative data. Pediatr Diabetes. 2012;13:229–34.

5. Klompas M, Eggleston E, McVetta J, Lazarus R, Li L, Platt R. Automated Detection and Classification of Type 1 Versus Type 2 Diabetes Using Electronic Health Record Data. Diabetes Care. 2013;36:914–21.

6. Lawrence JM, Black MH, Zhang JL, Slezak JM, Takhar HS, Koebnick C, et al. Validation of Pediatric Diabetes Case Identification Approaches for Diagnosed Cases by Using Information in the Electronic Health Records of a Large Integrated Managed Health Care Organization. Am J Epidemiol. 2014;179:27–38.

7. Zhong VW, Pfaff ER, Beavers DP, Thomas J, Jaacks LM, Bowlby DA, et al. Use of administrative and electronic health record data for development of automated algorithms for childhood diabetes case ascertainment and type classification: the SEARCH for Diabetes in Youth Study. Pediatr Diabetes. 2014;15:573–84.

8. Zhong VW, Obeid JS, Craig JB, Pfaff ER, Thomas J, Jaacks LM, et al. An efficient approach for surveillance of childhood diabetes by type derived from electronic health record data: the SEARCH for Diabetes in Youth Study. J Am Med Inform Assoc. 2016;23:1060–7.

9. Sharma M, Petersen I, Nazareth I, Coton SJ. An algorithm for identification and classification of individuals with type 1 and type 2 diabetes mellitus in a large primary care database. Clin Epidemiol. 2016;8:373–80.

10. Schroeder EB, Donahoo WT, Goodrich GK, Raebel MA. Validation of an algorithm for identifying type 1 diabetes in adults based on electronic health record data. Pharmacoepidemiol Drug Saf. 2018;27:1053–9.

11. Luk AOY, Ke C, Lau ESH, Wu H, Goggins W, Ma RCW, et al. Secular trends on incidence of type 1 and type 2 diabetes in Hong Kong. PLoS Med. 2020 (in press).
